# CD4^+^ to CD8^+^ T cell imbalance drives poor Achilles tendon repair in patients

**DOI:** 10.1016/j.isci.2025.114612

**Published:** 2026-01-02

**Authors:** Franka Klatte-Schulz, Sven Geißler, Nicole Bormann, Susann Minkwitz, Serafim Tsitsilonis, Sebastian Manegold, Tobias Gehlen, Josephine A. Melzer, Alper Kurtoglu, Aysha Bonell, Katharina Schmidt-Bleek, Georg N. Duda, Birgit Sawitzki, Britt Wildemann

**Affiliations:** 1 Julius Wolff Institute, Berlin Institute of Health at Charité-Universitätsmedizin Berlin, 13353 Berlin, Germany; 2 BIH-Center for Regenerative Therapies, Berlin Institute of Health at Charité-Universitätsmedizin Berlin, 13353 Berlin, Germany; 3 Center for Musculoskeletal Surgery, Charité-Universitätsmedizin Berlin, 13353 Berlin, Germany; 4 Sporthopaedicum Berlin, 10627 Berlin, Germany; 5 BG Unfallklinik Frankfurt Am Main, 60389 Frankfurt Am Main, Germany; 6 Move Ahead – FOOT ANKLE AND SPORTSCLINIC, 10117 Berlin, Germany; 7 Wyss Institute for Biologically Inspired Engineering, Harvard University, Boston, MA 02115, USA; 8 Center of Immunomics, Berlin Institute of Health at Charité-Universitätsmedizin Berlin, 13353 Berlin, Germany; 9 Institute of Medical Immunology, Charité-Universitätsmedizin Berlin, Corporate Member of Freie Universität Berlin and Humboldt University of Berlin, 13353 Berlin, Germany; 10 Experimental Trauma Surgery, Department of Trauma-, Hand- and Reconstructive Surgery, Jena University Hospital, Friedrich Schiller University Jena, 07747 Jena, Germany

**Keywords:** Immunology, Cell biology

## Abstract

Insufficient healing of the Achilles tendon remains a frequent clinical challenge, creating a need for early markers that identify patients at risk of impaired healing. To examine whether adaptive immunity contributes to these outcomes, we analyzed T cell subsets in blood and hematoma collected during surgery. Patients with a higher CD4^+^/CD8^+^ T cell ratio at surgery reported more pain, showed reduced functional recovery, and greater tendon strain after 12 months. Conversely, elevated CD8^+^ T cell levels, and the CD28-/CD57^+^ memory subset, coincided with more favorable outcomes. We then investigated how these cells affect tendon healing by co-culturing human tenocytes with CD4^+^ or CD8^+^ T cells. Exposure to CD4^+^ T cells increased collagen type 3, IL-17 receptors and matrix metalloproteinases expression, indicating a shift toward impaired extracellular matrix organization. These results suggest that the CD4^+^/CD8^+^ T cell balance may serve as a prognostic marker and that modulating CD4^+^ T cell activity or IL-17 signaling could improve tendon repair.

## Highlights

• CD4^+^/CD8^+^ T cell ratio at surgery predicts Achilles tendon healing outcome
• CD8^+^ memory T cell subsets associate with reduced tendon elongation
• CD4^+^ T cells drive IL-17-related catabolic responses in human tenocytes
• T cell profiling enables early identification of patients at risk for poor repair

## Graphical abstract

**Figure:**
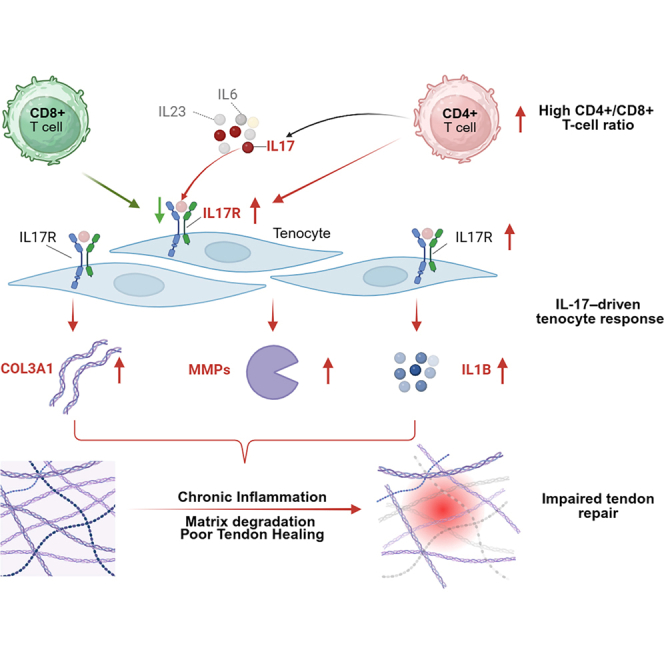

## Introduction

Tendon injuries represent one of the most common reasons for musculoskeletal consultations and the Achilles tendon is, with about 20 of 100,000 cases every year, the most affected tendon in the human body with raising incidence.^1^ In case of acute rupture, approximately 30% of the patients fail to heal properly, which particularly impedes professional and recreational athletes to return to sports activity.^2^^,^^3^ This unsatisfactory patient outcomes after Achilles tendon injury combines with high health care costs. Moreover, despite surgery itself and physiotherapeutic rehabilitation, there is to date no clinically approved treatment option to prevent impaired Achilles tendon healing or to improve the healing outcome after rupture. A fundamental understanding of the mechanisms leading to impaired tendon healing will not only allow identification of the patients most at risk but will also enable the possibility of developing targeted therapies.

Cells of the immune system regulate healing processes throughout the human body and lead to successful healing or, in case of an immune disbalance, can contribute to excessive inflammation or impaired healing responses. The contribution of immune cells in tendon healing has long been unrecognized and is still far from being understood. Inflammatory infiltrates have been observed in both acute tendon ruptures and chronic tendon lesions,^4^^,^^5^ suggesting that immune activation is potentially a common feature across different tendon pathologies. Studies so far have concentrated on the role of macrophages in tendon healing, showing the importance of timely regulated macrophage polarization for optimal tendon healing.^6^^,^^7^^,^^8^^,^^9^ Next to macrophages, other immune cells might also contribute to tendon healing and especially the role of cells of the adaptive immune system in tendon regeneration is still an emerging field of research.^10^ In contrast to cells of the innate immune system, cells of the adaptive immune system such as T cells and B cells exhibit an antigen-specific memory after exposure to a pathogen.^11^ In case of an injury, these cells might act through mediation of inflammatory cytokines and thus modulation of the inflammatory tendon environment or through direct interplay with resident tenocytes.^12^ Despite the presence and temporal dynamics of T cells in diseased Achilles tendons,^13^^,^^14^^,^^15^ understanding of the regulatory mechanisms of T cells in tendon pathophysiology remain unclear.^10^^,^^12^ Single cell transcriptomic analysis identified distinct T cell populations to be upregulated during tendon damage showing their involvement in the physiology of tendon injury and repair.^16^^,^^17^ Furthermore, using imaging mass cytometry, the tight proximity of T cells with tenocytes in rotator cuff tendinopathy was confirmed, underlining possible tendon immune cell interactions.^17^

For bone regeneration, it becomes clear that T cells are required for a successful regeneration process and that, in particular CD8^+^ T cells may be linked to delayed bone healing.^18^ In more detail, terminally differentiated memory T cells (CD3^+^CD8^+^CD11a^++^CD28^−^CD57^+^) were found in elevated amounts in patients with delayed bone healing and depletion of these T cells in a mouse model led to improved bone healing.^18^ In contrast, CD4^+^ regulatory T cells (Tregs) were associated with improved wound and bone healing.^19^^,^^20^ The CD4^+^ T cell phenotype highly depends on the microenvironment. CD4^+^ T helper cells include, in addition to the anti-inflammatory Treg and Th2 populations, pro-inflammatory subpopulations such as Th1 and Th17 cells. Particularly, Th17 cells as one of the main producers of interleukin-17 (IL-17),^21^^,^^22^^,^^23^ may regulate tendon healing. The isotype IL-17A is a pro-inflammatory cytokine that acts in a variety of inflammatory and autoimmune diseases through binding to the heterodimeric IL-17 receptor A and C (IL-17RA/IL-17RC) complex.^24^ These receptors are expressed on various cell types including endothelial cells, myeloid cells, and fibroblasts. Downstream effects mediated through IL-17A signaling include induction of cytokines, such as IL-1B, IL-6, IL-8, and tumor necrosis factor α (TNF-α), chemokines such as CXCL1, CXCL2, CCL20, and matrix metalloproteinase (MMP).^25^^,^^26^^,^^27^ Thus, IL-17-mediated inflammatory signaling may impair tendon healing due to Extracellular Matrix (ECM) degradation and loss of structural integrity. The contribution of IL-17A to early tendinopathy at the rotator cuff has previously been shown.^28^^,^^29^^,^^30^ Moreover, in chronic Achilles tendon enthesis of spondyloarthritis patients, the anti-IL-17A antibody secukinumab showed promising results regarding symptom relief in a phase 3 clinical trial.^31^

It remains unclear whether immune cells participate in the onset of tendon healing and, if so, which immune cells or their inflammatory mediators contribute to the success or failure of tendon regeneration. Therefore, we aimed to underscore the pivotal role of adaptive immunity, specifically CD4^+^ and CD8^+^ T cells as well as their terminally differentiated memory subsets (CD8^+^CD11a^++^CD28^−^CD57^+^), in the healing process of Achilles tendon ruptures. We hypothesize that T cells determine the healing of Achilles tendons after acute rupture and can be used as prognostic indicator of healing outcomes, as well as a potential target for more effective and personalized treatments of tendon injuries in the future.

## Results

### Experimental design and subgroup definition of patient study

**Figure 1:**
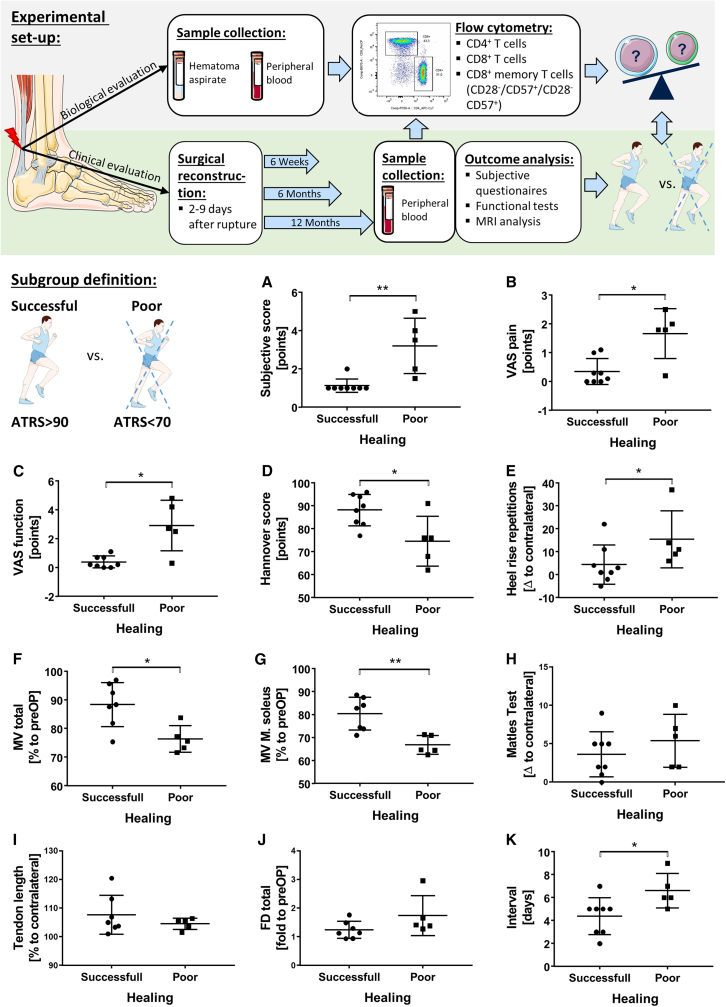
Experimental design and subgroup definition: Grouping of successful healing and poor healing patients according to the ATRS (>90, <70) (A) Subjective score: 0–6, with 6 being worst. (B and C) VAS pain/function: 0–10, with 10 being worst. (D) Hannover score: 0–100, with 100 being best. (E) Δ heel rise repetitions: measured as difference to the contralateral side. (F) Total MV: calculated as sum of the three muscles M. soleus, M. gastrocnemius lateralis/medialis, and given in percentage to the pre-operative state. (G) MV of solely M. soleus given in percentage to pre-operative state. (H) Matles test given as Δ of Achilles tendon resting angle in plantar flexion relative to the contralateral side. (I) Tendon length measured by MRI and given as percentage to contralateral side. (J) Fatty degeneration (FD) measured by MRI and given as fold to pre-operative value. (K) Interval: time from rupture to surgery. Values reflect *n* = 8 successful healer and *n* = 5 poor healer (biological replicates). For MRI-based parameters (MV, tendon length, and FD), values reflect *n* = 7 for successful healer. Mann-Whitney *U* test, Asterisk: ∗*p* ≤ 0.05 and ∗∗*p* ≤ 0.01. The experimental design image was adapted from Servier Medical Art (https://smart.servier.com/), licensed under CC BY 4.0 (https://creativecommons.org/licenses/by/4.0/). Top, experimental design; bottom, subgroup definition.

The purpose of this study was to explore the influence of adaptive immunity on the healing process of Achilles tendon injuries. We recruited 31 patients who had acute Achilles tendon rupture, of whom 26 patients completed the follow-up 12 months after surgery. While analyses were performed on the complete cohort of 26 patients with one-year follow-up, group comparisons in the main text focus on the two most distinct outcome groups (successful vs. poor healing), with results from the intermediate group provided mainly in the supplemental information. We obtained hematoma aspirate and longitudinal peripheral blood samples at different time points in all patients and determined the amount of various T cell subsets by flow cytometry. We also evaluated the clinical, functional and structural outcomes using different clinical scores and imaging metrics. The study design is illustrated in Figure 1.

### ATRS is a valid parameter to discriminate between successful and poor healers

The Achilles tendon total rupture score (ATRS) is a validated and suitable subjective patient-reported outcome measure for assessing the recovery from Achilles tendon injury.^32^^,^^33^ The ATRS evaluates aspects such as pain, function, and activity level. However, no clear threshold has been defined to distinguish between successful and poor healing. In this study, we defined the cutoff for poor healing as an ATRS <70 points, intermediate healing as an ATRS between 70 and 90 points, and successful healing as an ATRS >90 points and compared the ATRS with other clinical indicators of subjective and functional outcomes in patients at 12 months after surgery (Figures 1 and S1). While these cutoffs differ slightly from published patient acceptable symptom state (PASS) criteria, which range, for example, from 52 to 57 points at 12–24 months in a Danish cohort^34^ and approximately 75 points at 12–27 months in a Swedish cohort,^35^ they capture meaningful functional differences within our cohort. We found that patients with poor healing had lower subjective scores (*p* = 0.002), more pain (visual analog scale [VAS] pain: *p* = 0.016), perceived functional impairment (VAS function: *p* = 0.011) and performed fewer heel-raise repetitions (*p* = 0.048) than patients with successful healing according to their ATRS. Patients with poor healing also had lower Hannover score (*p* = 0.027), lower total muscle volume (muscle volume [MV] total: *p* = 0.030), and lower MV of the soleus muscle ([M. soleus] MV M. soleus: *p* = 0.006) compared to patients with successful healing (Figures 1A–1G). The scores for patients with intermediate healing lay in-between successful and poor healers (Figure S1). Achilles tendon elongation and the fatty degeneration of the corresponding muscles did not correlate with the ATRS (Figures 1H–1J). Interestingly, the time between rupture and surgery also influenced the healing outcome, with successful healers undergoing surgery earlier than poor healers (Figure 1K). Patient age and BMI did not affect the healing outcome (*p* = 0.33 and *p* = 0.22, respectively). This analysis indicates that the ATRS is a valid parameter to discriminate between successful and poor healers in terms of pain and function, but not in terms of tendon elongation, which should be evaluated separately. Tendon elongation seems to represent a distinct form of suboptimal healing that may occur without pronounced subjective impairment but still deviates from normal tendon anatomy and can affect long-term biomechanics.

### Immune cell profiling reveals early T cell involvement in tendon healing

In parallel with the clinical follow-up, we performed a detailed analysis of the immune cell composition in the hematoma aspirate and peripheral blood of all patients in our cohort. Table S1 summarizes the mean percentages of cell populations at different time points (pre-operative, intra-operative, 6 weeks, 6 months, and 12 months). We found that lymphocytes in peripheral blood were lower at surgery, increased over time, and showed a significant difference after 12 months (*p* = 0.044). CD3^+^ T cells were significantly higher in pre-operative peripheral blood (mean = 66.4%) than in hematoma aspirate (mean = 45.9%) (*p* < 0.001). The relative proportions of CD4^+^ and CD8^+^ T cells did not significantly differ between the hematoma and the peripheral blood, and systemic levels did not change significantly over time. However, certain subsets of effector memory CD8^+^ T cells (CD28^−^, CD57^+^, and CD28^−^CD57^+^) were lowest in pre-operative peripheral blood and highest in the hematoma aspirate and subsequent blood samples (*p* = 0.026 < 0.0001).

**Figure 2:**
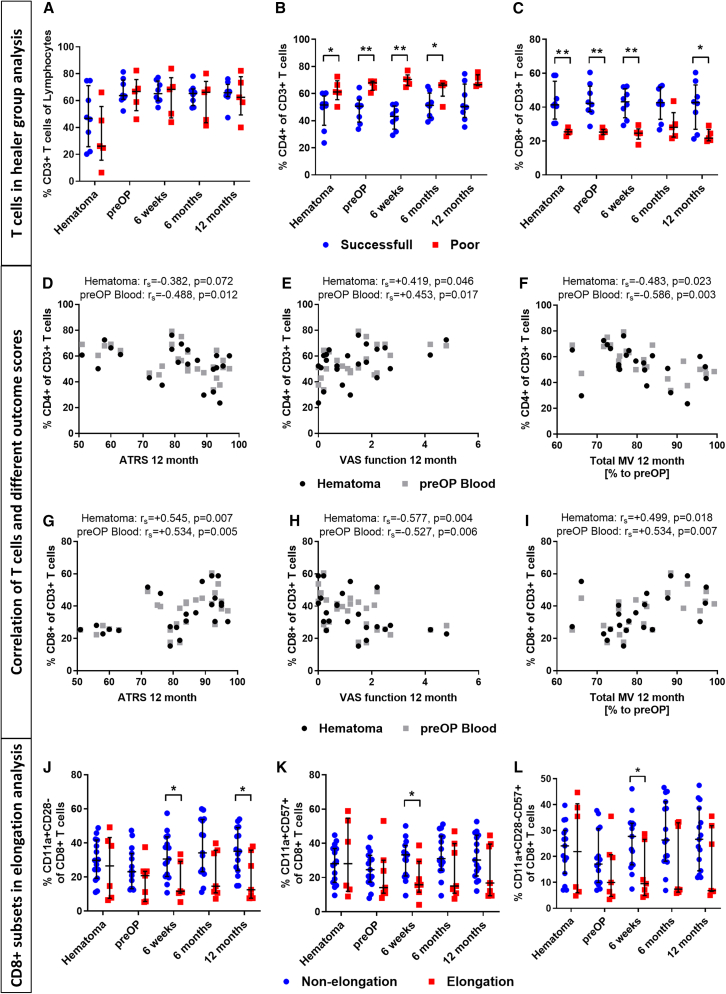
T cells in Achilles tendon healing outcome (A–C) (A) CD3^+^, (B) CD4^+^, and (C) CD8^+^ T cells in the successful healing (ATRS >90) and poor healing (ATRS <70) group after 12 months (D–F) CD4^+^ T cells in pre-operative peripheral blood and hematoma aspirate correlate to (D) ATRS 12 months after surgery (high = good) and (E) VAS function 12 months after surgery (high = bad), (F) total MV 12 months after surgery given as % to pre-operative total MV (high = good). (G–I) CD8^+^ T cells in hematoma aspirate correlates to (G) ATRS 12 months after surgery (high = good), (H) VAS function 12 months after surgery (high = bad), and (I) total MV 12 months after surgery given as % to pre-operative total MV (high = good). (J–L) CD8^+^ memory T cell subsets of (J) CD8^+^CD11a^++^CD28^−^, (K) CD8^+^CD11a^++^CD57^+^, and (L) CD8^+^CD11a^++^ CD57^+^CD28^−^ T cells in blood and hematoma aspirate of patients with and without tendon elongation (Matles <4, >7) after 12 months. Cell populations were measured by flow cytometry and given in percentage to the respective parent population. (A–C) *n* = 13 patients (*n* = 8 successful, *n* = 5 poor). Statistics: Mann-Whitney *U* test; ∗*p* ≤ 0.05 and ∗∗*p* ≤ 0.01. (D–I) *n* = 23–26 patients (biological replicates); Statistics, Spearman correlation; Rs, Spearman correlation coefficient. (J–L) *n* = 22 patients (*n* = 15 non-elongation, *n* = 7 elongation). Statistics, Mann-Whitney *U* test, ∗*p* ≤ 0.05.

We then correlated the clinical outcome of tendon healing with the T cell subsets in the blood and hematoma aspirate. We observed that the T cell subsets were strongly correlated with the functional healing outcomes at 12 months, suggesting that the individual immune profile may have a long-term impact on tendon healing. Specifically, we found that patients with a higher frequency of (CD3^+^) T cells in their hematoma aspirate had less Achilles tendon elongation (Matles test) at 12 months than those with lower frequencies (Table S2), indicating a beneficial role of T cells in tendon repair. However, when grouping patients defined on the basis to their ATRS into successful and poor healers no differences occurred (Figure 2A).

### CD4^+^ T cells correlate with impaired clinical and structural healing outcomes

Among the T cell subsets, CD4^+^ T cells emerged as key discriminators between successful and poor healing groups. The percentage of CD4^+^ T cells showed a negative correlation with the healing outcome. CD4^+^ T cell frequencies were significantly increased in the hematoma aspirate (*p* = 0.030), pre-operative peripheral blood (*p* = 0.006), as well as peripheral blood 6 weeks and 6 months after surgery (*p* = 0.002, *p* = 0.030 respectively) in patients of the poor healing group (ATRS < 70) (Figure 2B). Higher CD4^+^ T cell frequencies also correlated with worse subjective scores (*p* = 0.015–0.027), more pain (*p* = 0.015–0.040), impaired function (VAS function: *p* = 0.007–0.046), lower Hannover scores (*p* = 0.026), and more tendon elongation (Matles test: *p* = 0.005–0.021). In addition, patients with higher CD4^+^ T cell counts were able to perform fewer heel raises (*p* = 0.026) and had lower muscle calf circumference ([MCC]: *p* = 0.008–0.040), total MV (*p* = 0.003–0.023), and MV of the M. soleus (*p* = 0.039) (Figures 2D–2F and S2).

### CD8^+^ T cells and their memory subsets are associated with improved tendon healing

In contrast to CD4^+^ T cells, higher frequencies of total CD8^+^ and memory-type CD11 high-expressing (CD11a^++^) CD8^+^ T cells were associated with successful tendon healing. These cells were more abundant in the successful healing group (ATRS > 90), including hematoma aspirate (*p* = 0.002), pre-operative peripheral blood (*p* = 0.002), as well as blood samples at 6 weeks and 12 months after surgery (*p* = 0.031 and *p* = 0.030, respectively) (Figure 2C). Patients with higher frequencies of CD8^+^ T cells in their hematoma aspirate and peripheral blood not only had higher ATRS (*p* = 0.005–0.012) but also exhibited better subjective scores (*p* = 0.002–0.039), less pain (VAS pain: *p* = 0.029–0.040), improved function (VAS function: *p* = 0.004–0.010), as well as higher MCC (*p* = 0.036), total MV (*p* = 0.007–0.018), and MV of the M. soleus (*p* = 0.047) (Figures 2G–2I; Table S2). Moreover, higher percentages of circulating CD8^+^ T cells correlated with less tendon elongation (*p* = 0.002–0.029) and less fatty degeneration of the M. soleus (*p* = 0.047) (Table S2). CD4^+^ and CD8^+^ T cell frequencies for patients with intermediate healing lay in-between successful and poor healers (Figure S2). In addition, markers of chronic activation, such as increased CD57 expression and absence of CD28, were significantly higher on CD8^+^(CD11a^++)^ memory T cells in blood samples of subjects with less tendon elongation (*p* = 0.001–0.044) and lower pain and function scores (VAS pain: *p* = 0.041; VAS function: *p* = 0.026) (Figures 2J–2L; Table S2).

Taken together, these data suggest that the individual T cell profile of patients affects Achilles tendon healing. CD4^+^ T cells appear to impair healing, whereas CD8^+^ T cells and their memory subsets are associated with improved repair outcomes.

### Distinct T cell signatures serve as prognostic markers of tendon healing outcome

Next, we examined whether the specific CD4^+^ and CD8^+^ T cell subsets in peripheral blood could be a suitable marker to identify patients at risk of developing poor tendon healing (ATRS <70) or tendon elongation (Matles >7°). We used receiver operating characteristic (ROC) curves to establish the optimal thresholds for circulating CD4^+^ T cells, CD8^+^ T cells or the CD4^+^/CD8^+^ T cell ratio at the time of surgery and for circulating CD8^+^ memory T cell subsets at 6 weeks after surgery.

**Figure 3:**
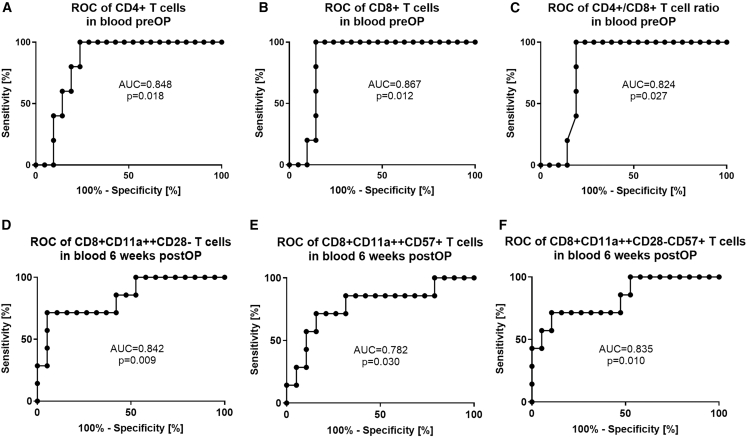
T cells as prognostic marker for Achilles tendon healing (A–C) (A) CD4^+^ of CD3^+^ T cells, (B) CD8^+^ of CD3^+^ T cells, and (C) CD4^+^/CD8^+^ T cell ratio in pre-operative peripheral blood. At a cutoff at ATRS±70 *n* = 5 poor healers and *n* = 21 healers were evaluated. (D–F) (D) CD11a^++^CD28^−^of CD8^+^ T cells, (E) CD11a^++^CD57^+^ of CD8^+^ T cells, and (F) CD11a^++^CD28^−^CD57^+^ of CD8^+^ T cells in peripheral blood 6 weeks after surgery. Cutoff Matles: ±7°: 26 patients (*n* = 7 tendon elongation and *n* = 19 non-elongation). Statistics: ROC analysis with 95% confidence interval, AUC, Area Under the Curve.

We found that in the pre-operative blood samples, a CD4^+^ T cell frequency of more than 59% of CD3^+^ T cells was associated with impaired Achilles tendon healing, with a sensitivity of 100% and a specificity of 76.2% (Area Under the Curve [AUC] = 0.848, *p* = 0.018). For CD8^+^ T cells, a frequency of less than 28% of CD3^+^ T cells was linked to impaired healing, with a sensitivity of 100% and a specificity of 85.7% (AUC = 0.867, *p* = 0.012). The CD4^+^/CD8^+^ T cell ratio was also predictive of impaired healing, with a threshold of more than 2.3 giving a sensitivity of 100% and a specificity of 81% (AUC = 0.848, *p* = 0.018) (Figures 3A–3C).

ROC analysis of CD8^+^ memory T cell subsets in the blood samples taken 6 weeks after surgery supported their prognostic value regarding tendon elongation. A CD28^−^memory T cell (CD8^+^CD11a^++^) level of less than 14% of CD8^+^ T cells was found as an optimal cutoff to predict Achilles tendon elongation, with a sensitivity of 71,4% and a specificity of 94,7% (AUC = 0.842, *p* = 0.009). For CD57^+^ memory T cells, a level of less than 20% of CD8^+^ T cells was determined as cutoff, which yielded 71.4% sensitivity and 84.2% specificity (AUC = 0.782, *p* = 0.030). Similarly, CD28^−^CD57^+^ memory T cells from CD8^+^ T cell fraction with less than 12% identified patients with Achilles tendon elongation, with a sensitivity of 71.4% and a specificity of 89.5% (AUC = 0.835, *p* = 0.010) (Figures 3D–3F).

In summary, ROC analysis shows that CD4^+^ and CD8^+^ T cells in pre-operative blood, as well as CD8^+^ T cell subsets in blood at 6 weeks post-surgery, can predict the healing outcome with high accuracy and enables the identification of patients at risk of poor healing or tendon elongation before or early after surgery.

### Co-culture with CD4^+^ T cells has detrimental effects on tenocytes and IL-17 polarization amplifies the effect

To better understand the causal relationship between individual T cell profile and clinical outcome of tendon repair, we investigated how CD4^+^ and CD8^+^ T cells modulate human tenocyte function *in vitro*. We focused on IL-17-producing CD4^+^ T cells, which are known to be involved in tendinopathy^28^^,^^29^ and may also affect healing in acute tendon rupture. In addition, we investigated the effects of CD8^+^ T cells, which have a known cytotoxic function but can also exert anti-inflammatory responses, for example, through their secretion of interferon-gamma (IFNG), a cytokine with pro- as well as anti-inflammatory potential.^36^^,^^37^

**Figure 4:**
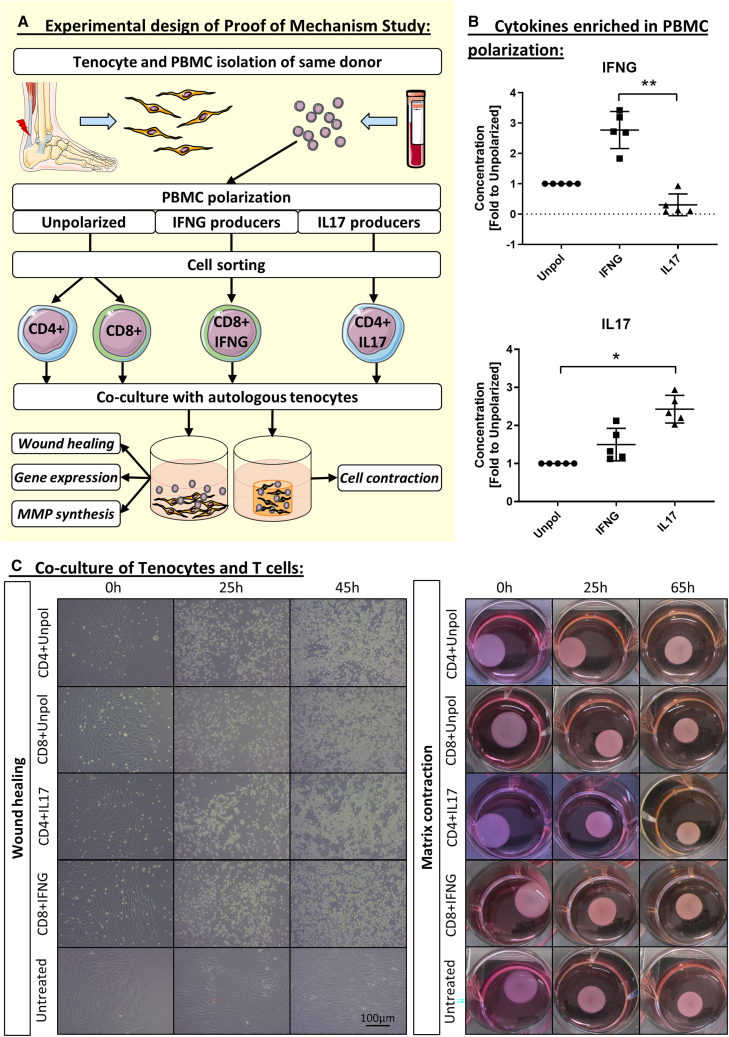
Proof of mechanism *in vitro* (A) Experimental setup. (B) Cytokines IFNG and IL-17 in PBMC supernatants after polarization. PBMCs were either left unpolarized (anti-CD3, anti-CD28 only), polarized into IFNG producers (anti-CD3, anti-CD28, anti-IL-4, IL-12, and IL-2), or polarized into IL-17 producers (anti-CD3, anti-CD28 anti-IFNG, IL-6, IL-23, IL-1B, and Transforming Growth Factor beta [TGFB]). Concentrations analyzed by enzyme-linked immunosorbent assay (ELISA) and given as fold to unpolarized PBMCs. (C) Exemplary images of tenocytes/T cells co-culture in wound healing assay and matrix contraction assay. Wound healing assay shows adherent tenocytes with a few T cells directly after co-culture (0 h) and strong T cell proliferation until 45 h of co-culture. Scale bars, 100 μm (length). Matrix contraction shows tenocytes and T cells in 3D co-culture in collagen gels in a 12-well plate directly after seeding and polymerization (0 h) and its decrease in size (surface area) until 65 h of incubation. *n* = 5 biological replicates (B and C). Statistics, Friedman test with Dunn’s multiple comparison, ∗*p* ≤ 0.05 (∗), ∗∗*p* ≤ 0.01. The images in (A) were adapted from Servier Medical Art (https://smart.servier.com/), licensed under CC BY 4.0 (https://creativecommons.org/licenses/by/4.0/).

We isolated tenocytes from patients with Achilles tendon ruptures and cultured them together with their autologous CD4^+^ or CD8^+^ T cells from peripheral blood mononuclear cells (PBMCs) that were either unpolarized or polarized toward an IL-17-producing (IL-17-polarized) and IFNG-producing (IFN-polarized) phenotype (Figure 4A). The successful polarization of CD4^+^ and CD8^+^ T cells was confirmed by the significant increase in their IFNG and IL-17 secretion (Figure 4B). In 2D co-culture, T cells from all groups proliferated rapidly over 45 h and formed close contacts with their autologous tenocytes (Figure 4C).

**Figure 5:**
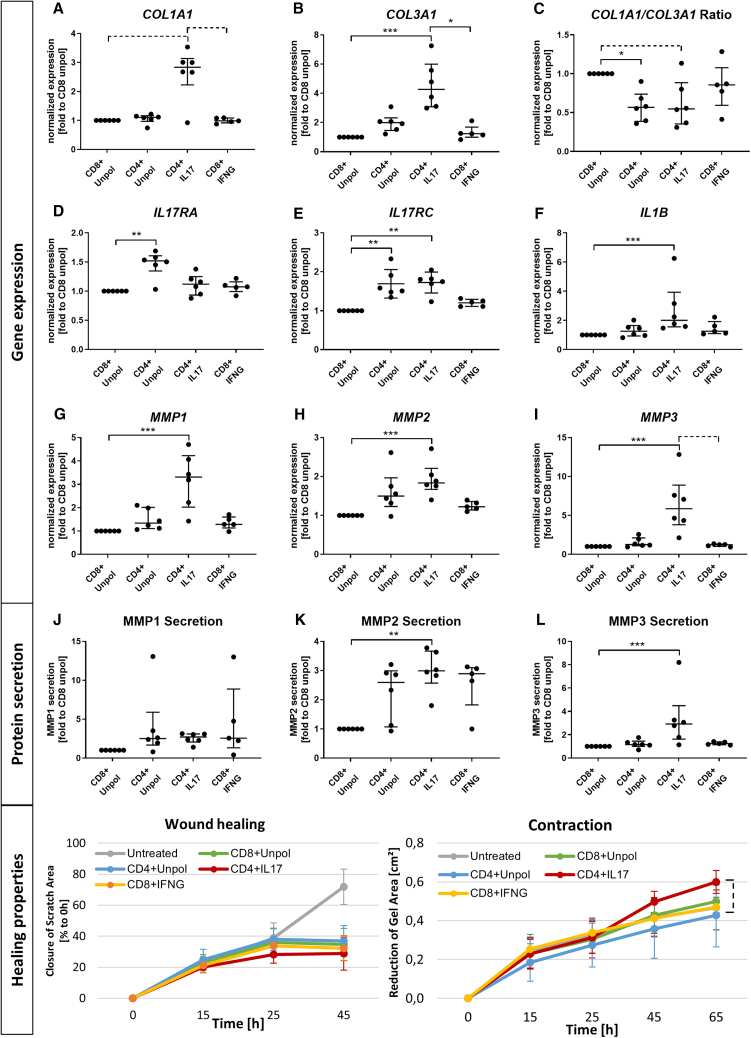
Gene expression, protein secretion, and healing properties of tenocytes kept in co-culture with autologous T cells Tenocytes were co-cultured with unpolarized CD8^+^ or CD4^+^ T cells, IL-17-polarized CD4^+^ T cells, or IFN-polarized CD8^+^ T cells in the respective stimulation medium (which did not contain exogenous IL-17 or IFNG). (A–I) Gene expression calculated as normalized expression to the housekeeping gene HPRT and given as fold to unpolarized CD8^+^ T cells (*n* = 5–6 biological replicates). (J–L) Protein secretion given as fold to unpolarized CD8^+^ T cells (*n* = 5–6 biological replicates). (M) Wound healing given as percentage of closure to the 0 h’ time point. (*n* = 6 biological replicates). (N) Contraction given as reduction of the size of the gel area in cm^2^ normalized to the 0 h’ time point (*n* = 5–6 biological replicates). Statistics, Kruskal-Wallis test and Dunn’s multiple comparisons test; ∗*p* ≤ 0.05, ∗∗*p* ≤ 0.01, ∗∗∗*p* ≤ 0.001. A dashed significance bar indicates a trend (p ≤ 0.1)

We then measured the gene expression level of collagen type 1 and 3 (*COL1A1* and *COL3A1*), IL-17 receptors (*IL-17RA* and *IL-17RC*), *IL-1B*, and *MMPs* (*MMP1/2/3*) in tenocytes, as well as their MMP secretion, wound healing, and contractility in collagen gels. The COL1A1/COL3A1 ratio in tenocytes was significantly influenced by the type and polarization of the co-cultured T cells. Tenocytes co-cultured with CD4^+^ T cells from IL-17-polarized PBMCs showed slightly higher *COL1A1* expression, highly increased *COL3A1* expression, and a subsequently lower *COL1A1*/*COL3A1* ratio than those co-cultured with unpolarized CD8^+^ T cells or IFN-polarized CD8^+^ T cells (*p* = 0.0003–0.086). Tenocytes co-cultured with unpolarized CD4^+^ T cells showed also a decreased *COL1A1/COL3A1* ratio compared to their CD8^+^ counterparts (*p* = 0.036) (Figures 5A–5C). The expression of IL-17 receptors (*IL-17RA* and *IL-17RC*) in tenocytes was also influenced by the co-culture conditions. The expression of *IL-17RC* was significantly higher in tenocytes co-cultured with both unpolarized and IL-17-polarized CD4^+^ T cells than in corresponding co-cultures with unpolarized CD8^+^ T cells (*p* = 0.003, *p* = 0.002, respectively). The expression of *IL-17RA* was increased only in co-cultures with unpolarized CD4^+^ T cells (*p* = 0.007) (Figures 5D and 5E). We also found that IL-17-polarized CD4^+^ T cells induced higher expression of *IL-1B* in tenocytes than unpolarized CD8^+^ T cells (*p* = 0.003) (Figure 5F). We observed that gene expression and protein secretion of MMPs involved in IL-17 signaling were increased in tenocytes co-cultured with CD4^+^ T cells, particularly upon IL-17 polarization, compared to CD8^+^ T cells (*p* = 0.0006–0.01) (Figures 5G–5L), whereas TIMP1 was unregulated. Then wound healing and matrix contractility of tenocytes co-cultured with T cells was examined. We found that co-culture with all T cell subsets reduced tenocyte wound healing ability compared to their monoculture (untreated) and the lowest wound healing capacity was observed in the IL-17-polarized CD4^+^ T cell group. Matrix contractility was lowest in co-culture with unpolarized CD4^+^ T cells and highest in co-culture with IL-17-polarized CD4^+^ T cells. However, these differences between T cell groups did not fully reach statistical significance (*p* = 0.092) (Figures 5M–5N). Taken together, these data showed that unpolarized CD4^+^ T cells had a different impact on tenocytes than CD8^+^ T cells, suggesting that CD4^+^ T cells may affect tendon healing. Furthermore, IL-17 polarization of CD4^+^ T cells in a stimulating environment enhanced the deleterious effects of CD4^+^ T cells on tenocytes, whereas IFN-polarized CD8^+^ T cells had similar effects on tenocytes as compared to unpolarized CD8^+^ T cells.

In complementary experiments, tenocytes were stimulated with conditioned medium (CM) from polarized and unpolarized T cells. CM from IL-17-polarized T cells had a similar, albeit less pronounced, effect on tenocyte gene expression than direct co-culture. This suggests that modulation of tenocytes is primarily cytokine-mediated, but also contact-dependent (Figure S3).

## Discussion

The findings from our study underscore the pivotal role of adaptive immunity, specifically CD4^+^ and CD8^+^ T cells, in the healing process of Achilles tendon ruptures. This research provides novel insights into the differential impact of these T cell subsets on tendon healing outcomes, thus offering potential avenues for novel prognostic approaches and therapeutic intervention.

Our data reveal that a higher CD4^+^/CD8^+^ T cell ratio at the time of surgery is associated with poorer tendon healing outcomes, as evidenced by increased pain, decreased function, and greater tendon elongation. Conversely, higher levels of CD8^+^ T cells correlate with better healing, suggesting these cells play a positive role in tendon repair. The predictive value of these T cell ratios was confirmed through receiver operating characteristic (ROC) analysis. A CD4^+^ T cell frequency exceeding 59% or a CD8^+^ T cell frequency below 28% in pre-operative blood samples were strong indicators of compromised healing. Similarly, specific CD8^+^ memory (CD11a^++^CD28^−^/CD57^+^/CD28^−^CD57^+^) T cell subsets measured in the peripheral blood at six weeks post-surgery were predictive of less tendon elongation after 12 months, highlighting the potential of these immune markers in the early identification of at-risk patients (Table S2). These findings contrast with the perceived role of CD4^+^ and CD8^+^ T cell subpopulations in the regeneration of other musculoskeletal tissues.^18^^,^^38^^,^^39^^,^^40^ Specifically, CD8^+^CD28^−^CD57^+^ memory T cells seem to exert a detrimental effect on bone healing.^18^ Given the distinct healing mechanisms of tendon and bone, especially concerning scar formation, we proposed that these cell populations may have varying impacts on acute Achilles tendon healing.^41^ This assumption is supported by previous research findings, which indicate that CD8^+^ T cells interfere with the fixation of implants in cancellous bone, but do not impair the healing of the Achilles tendon in a rat model.^42^

Based on our patient data, we assumed that CD4^+^ T cells, known for their potent IL-17 production,^21^^,^^22^^,^^23^ may exert a negative influence on tendon healing via the IL-17 signaling cascade. Previous studies have implicated IL-17A in the pathomechanism of tendinopathy of the rotator cuff,^28^^,^^29^ yet its role in acute Achilles tendon ruptures remains elusive. We propose that stress-induced lymphopenia resulting from Achilles tendon rupture may account for the reduced percentage of CD4^+^ T cells observed in patients exhibiting better tendon healing outcomes. However, the reliance on relative percentages of T cells hinders the verification of this hypothesis. Nevertheless, the continuous increase in overall lymphocyte percentages from pre-operative to 12 months post-operative time point supports our assumption. Given that CD4^+^ T cells, particularly Th17 cells, are potent IL-17 producers,^21^^,^^22^^,^^23^ their reduced presence during tendon healing may contribute to improved outcomes for patients.

To further investigate the impact of different immune cell subsets on Achilles tendon healing and to gain mechanistic insights into their effects, we directly co-cultured primary human tenocytes with various T cell subsets of the same patient. Our *in vitro* co-culture experiments revealed that CD4^+^ T cells, particularly under IL-17-polarized conditions, negatively impact tenocyte function by upregulating the expression of IL-17 receptors (*IL-17RA* and *IL-17RC*). This receptor upregulation in tenocytes potentially amplifies the effects of IL-17 and thus likely contributes to a loss of tendon ECM quality and chronic inflammation, which is detrimental to tendon integrity and repair. We observed in tenocytes stimulated by CD4^+^ T cells compared to CD8^+^ T cells a marked upregulation of matrix metalloproteinases (MMP1, MMP2, and MMP3) and pro-inflammatory cytokines (e.g., *IL-6* and *IL-1B*), which are downstream mediators of the IL-17 signaling cascade.^26^^,^^29^ This is in line with earlier reports showing elevated pro-inflammatory cytokine expression in tenocytes co-cultured with activated CD3^+^ T cells.^17^ These elevated cytokine levels may contribute to an increased initial inflammatory state that can exacerbate tissue damage and delay the onset of healing. The combined effect of this pro-inflammatory milieu and increased MMP activity leads to a feedback loop that perpetuates inflammation and fosters tissue damage. CD4^+^ T cells showed enhanced effects compared to CD8^+^ T cells in all experiments, highlighting their potential dominance in initiating and maintaining inflammatory responses at the tendon rupture side. Accordingly, our *in vitro* experiments have also shown that IL-17-polarized CD4^+^ T cells in particular impede wound healing and increase matrix contractility. This indicates that IL-17 affects tenocytes primarily not only through released cytokines but also via direct interaction with T cells, which appears to enhance the overall response.

One of the decisive factors for the healing of tendons is the capability to reconstitute the ECM. In particular, an increase in COL3A1 or the COL1A1/COL3A1 ratio is associated with lower biomechanical competence of tendons.^43^ Our *in vitro* experiments showed that upregulation of IL-17 receptor expression in tenocytes is also associated with an increase in *COL3A1* expression, especially in tenocytes co-cultured with CD4^+^ T cells under all polarization conditions. Previous studies have shown that IL-17A induces *COL3A1* expression in human tenocytes^28^ and that tenocytes co-cultured with activated CD3^+^ T cells tend to have a COL3A1 phenotype.^17^ The changes in *COL1A1* and *COL3A1* expression in tenocytes grown in IL-17-polarized CD4^+^ T cell co-cultures could be partly influenced by TGFB and IL-6 in the polarization medium^44^^,^^45^^,^^46^ but may also reflect the inflammatory milieu at the tendon rupture side, especially under poor healing conditions. Therefore, elevated CD4^+^ T cell levels could promote a shift toward a COL3A1 phenotype that reduces the biomechanical competence of the Achilles tendon and impairs the healing process.

In contrast, CD8^+^ T cells, particularly those producing IFNG, had less deleterious effects on tenocytes. Co-cultures with CD8^+^ T cells did not increase the expression of pro-inflammatory cytokines, MMPs, or *COL3A1* in tenocytes, suggesting a protective role in maintaining ECM integrity and promoting a more favorable healing environment. Accordingly, a recent study has shown that human effector CD8^+^ T cells not only mediate cytotoxicity, but also promote tissue remodeling.^47^ On the other hand, an alternative interpretation of our results must consider that COL3A1 is associated with increased tendon elasticity, suggesting a regenerative pathway rather than solid scarring, which is primarily dominated by COL1A1.^48^ CD8^+^ T cells may promote increased COL1A1 expression in tenocytes, leading to solid scarring at the injury side and improved biomechanical healing at 12 months. Conversely, CD4^+^ T cells may foster prolonged regenerative processes via COL3A1 but result in poorer tendon biomechanical competence at the same time point. Therefore, the quality of the formed (scar) tissue is crucial for the long-term biomechanical capacity of the Achilles tendon. This aspect could not be investigated in this study and requires further detailed analysis, which we will address in future research.

Our improved understanding of the distinct roles of CD4^+^ and CD8^+^ T cells in tendon healing opens up several therapeutic avenues. Targeting IL-17-producing CD4^+^ T cells or their effects on tenocytes could be a promising strategy to reduce inflammation and ECM degradation. This could be achieved through IL-17 neutralizing antibodies, which mitigate its pro-inflammatory effects, thereby preserving ECM integrity and promoting better healing outcomes.^28^^,^^49^ Inhibiting the interaction between IL-17 and its receptors on tenocytes could prevent the downstream inflammatory cascade.^50^^,^^51^ Strategies to reduce the recruitment or activation of detrimental CD4^+^ T cell subsets while enhancing the beneficial CD8^+^ T cell responses could optimize the immune environment for healing.^52^^,^^53^^,^^54^ Moreover, the identification of T cell ratios and specific memory T cell subsets as prognostic markers can inform personalized treatment plans. Patients identified as being at higher risk for poor healing could receive tailored interventions aimed at modulating their immune responses, potentially improving their clinical outcomes. Given that our tendon samples were derived from patients during the early proliferative phase of tendon healing, IL-17 blockade is expected to be particularly beneficial at this stage by limiting excessive inflammation and fibrotic signaling, thereby promoting more organized extracellular matrix deposition and improved tendon healing. In addition, our results also show that the timing of the operation has a significant influence on the healing of the tendon. The later the surgery was performed, the worse the ATRS (Achilles tendon Total Rupture Score) at follow-up. This is consistent with other studies reporting that longer time intervals between Achilles tendon rupture and surgical repair are associated with worse functional outcomes and higher risk of tendon elongation.^55^^,^^56^ In a previous study, we were able to show that the functional results were worse in patients who were operated on within 48 h of the rupture than in patients who were operated on after 48 h, even though the same surgical technique and rehabilitation program were used.^57^ This indicates that there is an optimal time window for Achilles tendon surgery, which is after 48 h, but ideally no later than 5 days after the rupture.

Our study highlights several important considerations for future research. First, the specific contributions of various CD4^+^ T cell subpopulations, such as Th1, Th2, Th17, and Tregs, to tendon healing need to be explored in more detail. These subsets are known to have distinct roles during tissue repair. Th1 and Th17 cells typically promote matrix degradation through pro-inflammatory cytokine release, whereas Th2 and Tregs have been linked to the resolution of acute inflammation.^58^^,^^59^^,^^60^^,^^61^ Understanding how this balance is regulated within the tendon microenvironment will be essential for clarifying the contribution of adaptive immune responses to tendon regeneration. Additionally, investigating the interactions between T cells and other immune cells, such as macrophages and B cells, in the context of tendon healing could provide a more comprehensive understanding of the immune mechanisms involved. Longitudinal studies are also necessary to evaluate the long-term effects of immune modulation on tendon structure and function, ensuring that therapeutic interventions do not compromise the durability of the repair. While our data were obtained from patients with acute Achilles tendon rupture, we assume that T cells and their mediators also play an important role in the healing of other tendon tissues, as suggested by the reported contribution of IL-17A to rotator cuff tendinopathy.^17^^,^^28^^,^^29^^,^^30^ Furthermore, the impact of the CD4^+^/CD8^+^ T cell ratio is likely to vary depending on the type of tendon pathology. Acute injuries and chronic degenerative conditions differ substantially in their inflammatory environment, tissue structure, and healing dynamics, which may influence how T cells modulate repair processes. This has been summarized for the roles of innate and adaptive immune cells in chronic versus acute tendon pathologies.^12^ In addition, characteristics of the patient cohort, including age, comorbidities, and activity level, may further affect the relationship between T cell ratios and tendon healing outcomes.^62^ Therefore, confirmation in other tendon types and patient populations will be required to determine the extent of its applicability.

Taken together, our findings open new avenues for therapeutic intervention that target immune responses to improve tendon repair outcomes. However, further translational and clinical research will be required to determine how these insights can be effectively implemented in patient care.

### Limitations of the study

Several limitations should be considered when interpreting the findings of this study. First, the sample size of the clinical study was modest, reflecting the number of eligible patients treated during two-year recruitment period at a large tertiary trauma center. At the time of patient inclusion, it was not possible to predict individual healing outcomes or subsequent allocation to outcome groups. For this reason, no formal power calculation was performed. Although the cohort was sufficient to demonstrate significant associations between T cell profiles and clinical outcome, larger cohorts will be required to validate the proposed prognostic threshold and to assess additional covariates with adequate statistical power. Second, MRI-based assessment may provide a more direct and quantitative measure of tendon elongation, but MRI data were not available for all patients at all time points. Therefore, the Matles test was used as a consistent and validated clinical measure across the entire cohort. When interpreting our MRI-based results, it should also be noted that comparisons were made with pre-operative scans rather than with the contralateral side. Early atrophic or edematous changes in the muscle may have already influenced the baseline values and affected our evaluation. Third, this study focused on relative rather than absolute frequencies of T cell subsets, which might provide additional information on their biological relevance. Our analyses were restricted to T cells, and complementary multi-omic or cytokine-level profiling of other immune mediators could help to further define the role of immune responses in tendon healing. Unfortunately, no additional biological material from this cohort was available for such analyses, which should therefore be addressed in future studies.

Further, the *in vitro* co-culture experiments cannot fully reproduce the complex *in vivo* environment of tendon repair, including mechanical loading, vascularization, or contributions from additional immune and stromal cell populations. The use of polarized T cell subsets provides mechanistic insight, but the degree to which these *in vitro* conditions reflect the inflammatory milieu at the rupture site remains uncertain. Furthermore, the polarization media contained cytokines (e.g., IL-6, TGFB, and IL-1B) that may partially influence tenocyte gene expression, although the observed effects were consistent with known IL-17-mediated pathways. Future work should also focus on characterizing the local molecular and cellular microenvironment throughout the entire healing process in humans, as this will be a key to understanding how immune-tenocyte interactions and cytokine signaling dynamically shape tendon regeneration over time.

Finally, sex-based analyses could not be performed. This was because the clinical cohort included only three women and the *in vitro* cohort consisted exclusively of men. Therefore, the study was underpowered to detect sex-related differences and potential sex-dependent effects on tendon healing or T cell phenotypes must be considered unresolved. Further studies with more balanced cohorts are required to establish whether the immune signatures identified are affected by sex, gender, age or other patient-specific factors.

## Resource availability

### Lead contact

Further information and requests for resources and reagents should be directed to and will be fulfilled by Dr. Franka Klatte-Schulz (franka.klatte@bih-charite.de).

### Materials availability

This study did not generate new unique reagents.

### Data and code availability

• Data reported in this paper will be shared by the lead contact upon request. Raw and processed data supporting the figures (including those in the supplemental information) have been deposited in Mendeley Data and are publicly available at https://doi.org/10.17632/5ykrbhjf4v.1 (https://data.mendeley.com/datasets/5ykrbhjf4v/1).
• This paper does not report original code.
• Any additional information required to reanalyze the data reported in this paper is available from the lead contact upon request.

## Acknowledgments

We would like to acknowledge the assistance of the BIH Cytometry Core Facility for Flow Cytometry measurements and T cell sorting for the *in vitro* study as well as BIH Service Unit “Cell Harvesting” for supporting sample logistics. We furthermore acknowledge Dr. Erik Brauer for support in the methodology for matrix contraction assay. The graphical abstract was created with BioRender.com and is available under Geiβler, S. (2026), https://BioRender.com/31ay95. This work was supported by 10.13039/501100002347Federal Ministry of Education and Research (BMBF) through funding of 10.13039/501100017268BIH Center for Regenerative Therapies (FKZ1315848A) and the 10.13039/501100001659German Research Foundation (DFG) through funding of the SFB 1444. Additional support was provided by the Gender Equality Fund of the 10.13039/501100017268Berlin Institute of Health. Funding from the 10.13039/100020655European Health and Digital Executive Agency (HADEA) to the PROTO Consortium (grant no. 101095635) is gratefully acknowledged.

## Author contributions

Conceptualization, F.K.-L., S.G., S.M.A., B.S., and B.W.; data curation, F.K.-L., S.G., B.S., and B.W.; formal analysis, F.K.-L., and S.G.; methodology, F.K.-L., S.G., T.G., N.B., S.M.I., S.T., S.M.A., K.S.-B., and B.S.; investigation, F.K.-L., N.B., S.M.I., J.A.M., A.K., and A.B.; visualization, F.K.-L., and S.G.; funding acquisition, F.K.-L., S.G., and B.W.; supervision, G.N.D., S.T., S.M.A., B.S., and B.W.; writing – original draft, F.K.-L., S.G., and T.G.; writing – review and editing, F.K.-L., S.G., N.B., S.T., S.M.A., T.G., K.S.-B., G.N.D., B.S., and B.W.

## Declaration of interests

F.K.-L., S.G., S.T., S.M.A., T.G., K.S.-B., G.N.D., B.S., and B.W. have submitted a patent application WO2024002914A1, “Prediction of, and composition to improve, tendon healing” on the basis of this work.

## Declaration of generative AI and AI-assisted technologies in the writing process

During the preparation of this work, the authors used DeepL Write and DeepL Translate to improve the language. After using these tools, the authors reviewed and edited the content as needed and take full responsibility for the content of the publication.

## STAR★Methods

### Key resources table

**Table:** 

REAGENT or RESOURCE	SOURCE	IDENTIFIER
**Antibodies**		

Anti-human CD3 (clone UCHT1)	BD Biosciences	Cat# 555335; RRID: AB_395750
Anti-human CD4 (clone RPA-T4)	BD Biosciences	Cat# 555346; RRID: AB_398613
Anti-human CD8 (clone RPA-T8)	BD Biosciences	Cat# 555369; RRID: AB_395860
Anti-human CD11a (clone HI111)	BD Biosciences	Cat# 555379; RRID: AB_395871
Anti-human CD28 (clone CD28.2)	BD Biosciences	Cat# 555726; RRID: AB_396018
Anti-human CD57 (clone NK-1)	BD Biosciences	Cat# 555619; RRID: AB_395984
Anti-human CD19 (clone HIB19)	BD Biosciences	Cat# 555412; RRID: AB_395791
Anti-human CD45 (clone HI30)	BD Biosciences	Cat# 555482; RRID: AB_395864
Anti-human CD3 (clone OKT3)	BioLegend	Cat# 317328; RRID: AB_2562907
Anti-human CD4 (clone OKT4)	BioLegend	Cat# 317410; RRID: AB_571955
Anti-human CD8 (clone SK1)	BioLegend	Cat# 344742; RRID: AB_2566513
Anti-human CD45 (clone J33)	Beckman Coulter	Cat# A74763; RRID: AB_2888654
Anti-human TCRgd (clone B1)	BioLegend	Cat# 331208; RRID: AB_1575108
Live/Dead Fixable Viability Dye	Invitrogen	Cat# L34973
Live/Dead Fixable Viability Dye	Invitrogen	Cat# L34955

**Biological samples**		

Human Achilles tendon rupture tissue	Center for Musculoskeletal Surgery (CMSC), Charite Universitätsmedizin Berlin, 13353 Berlin	N/A
Peripheral blood and hematoma aspirate	CMSC, Charite Universitätsmedizin Berlin, 13353 Berlin	N/A
Primary human tenocytes	CMSC, Charite Universitätsmedizin Berlin, 13353 Berlin	N/A
Patient-derived PBMCs	CMSC, Charite Universitätsmedizin Berlin, 13353 Berlin	N/A

**Chemicals, peptides, and recombinant proteins**		

Recombinant human IL-6	Peprotech	Cat# 200-06
Recombinant human IL-1β	Peprotech	Cat# 200-01 B
Recombinant human TGF-β1	Peprotech	Cat# 100-21
Recombinant human IL-23	R&D Systems	Cat# 1290-IL
Recombinant human IL-12p70	Peprotech	Cat# 200-12
Recombinant human IL-2	Novartis	N/A
Anti-human IFN-γ antibody	BD Biosciences	Cat# 554698; RRID: AB_395516
Anti-human IL-4 antibody	BioLegend	Cat# 500804; RRID: AB_315123
Collagenase (0.3%)	Sigma-Aldrich	Cat# C9407
Collagen Type I (rat tail)	Corning	Cat# 354236
Red Blood Cell Lysis Buffer (RBL)	eBioscience	Cat# 00-4300-54

**Critical commercial assays**		

DuoSet ELISA for human IFN-γ	R&D Systems	Cat# DY285
DuoSet ELISA for human MMP-1	R&D Systems	Cat# DY901
DuoSet ELISA for human MMP-2	R&D Systems	Cat# DY902
DuoSet ELISA for human MMP-3	R&D Systems	Cat# DY513
DuoSet ELISA for human TIMP-1	R&D Systems	Cat# DY970
DuoSet ELISA for human IL-17 A	R&D Systems	Cat# DY317

**Deposited data**		

Raw and processed data	Mendeley	Mendeley Data: https://doi.org/10.17632/5ykrbhjf4v.1 (https://data.mendeley.com/datasets/5ykrbhjf4v/1)

**Experimental models: Cell lines**		

Primary human tenocytes	Primary human cells from patients	N/A
Human PBMCs	Primary human cells from patients	N/A

**Oligonucleotides**		

PCR primers for qPCR	see supplemental information (Table S4)	N/A

**Software and algorithms**		

GraphPad Prism v7.0	GraphPad Software	https://www.graphpad.com
FlowJo v10	BD Biosciences	https://www.flowjo.com
ImageJ (Fiji)	NIH	https://imagej.nih.gov/ij/
OsiriX Lite v7.5.1	Pixmeo SARL	https://www.osirix-viewer.com
3D Slicer v4.6	Slicer Community	https://www.slicer.org
Tecan Spark Control Software	Tecan	https://www.tecan.com
Primer3 Software	Freeware	https://primer3.org

**Other**		

SepMate-50 tubes	STEMCELL Technologies	Cat# 85450
Lymphoprep	STEMCELL Technologies	Cat# 07861
X-Vivo 15 medium	Lonza	Cat# 04-418Q
DMEM/Ham’s F12 medium	Sigma-Aldrich	Cat# D8437
Fetal Calf Serum (FCS)	Sigma-Aldrich	Cat# F7524
Heparin & EDTA blood collection tubes	Sarstedt	N/A
BD FACSCanto II flow cytometer	BD Biosciences	N/A
BD FACSAria II cell sorter	BD Biosciences	N/A
Tecan SPARK plate reader	Tecan	N/A

### Experimental model and study participant details

#### Human study participants

Clinical study cohort (Achilles tendon rupture patients): To understand the critical role of patients’ immune status on tendon healing outcome in terms of pain, functional scores and MRI assessment, we examined adaptive T cells in peripheral blood and hematoma aspirate at the time of surgery and in follow-up up to 1 year after the operation. Patient recruitment and sample collection were conducted between December 2014 and January 2017.

This study included 31 patients with acute Achilles tendon rupture who were recruited at the Center for Musculoskeletal Surgery, Charité–Universitätsmedizin Berlin. All participants were adults and received a minimal invasive Achilles tendon reconstruction 2 to 9 days after rupture. Five patients were lost to follow-up and excluded from outcome-related analyses. Thus, 26 patients completed the 12-month follow-up and were included in correlations between immune parameters and clinical outcome. All procedures involving human participants were conducted in accordance with the Declaration of Helsinki and relevant institutional and national guidelines and regulations. The study protocol was approved by the Ethics Committee of Charité–Universitätsmedizin Berlin (approval number EA2/074/14). Written informed consent was obtained from all participants prior to inclusion.

Peripheral blood samples were collected pre-operatively, intra-operatively, and at 6 weeks, 6 months, and 12 months after surgery. Intra-operative hematoma aspirates were obtained at the rupture site during surgical repair. No randomization was performed, as this was an observational cohort study. Participants were allocated to outcome groups post hoc based on their Achilles tendon Total Rupture Score (ATRS) at 12 months after surgery: poor healing (ATRS <70), intermediate healing (ATRS 70–90), and successful healing (ATRS >90), as described in the results section. Sample sizes for each analysis are reported in the corresponding figure legends.

Participant age (mean: 39.4 ± 10.0 years), sex (3 females, 28 males), body mass index (BMI, mean: 24.8 ± 2.3), time interval between rupture and surgery (mean 4.9 ± 1.7 days), and sports activity level were recorded. Twenty-five participants were recreational athletes, five did not participate in sports regularly, and one was a professional athlete. Severe illness (HIV, Hepatitis) or ongoing medication known to affect immune or tendon function (systemic cortisone, anabolic steroids) were predefined exclusion criteria. Due to cohort composition, sex-based analyses were not statistically powered. Gender identity, race, ethnicity, and ancestry were not collected for this cohort and therefore could not be analyzed.

#### Human primary cells

Primary human tenocytes were isolated from Achilles tendon tissue obtained during surgical repair from additional patients not included in the clinical cohort. For *in vitro* experiments, tenocytes were derived from six male donors (mean age: 36.3 years; range: 24-50 years; 797 BMI: 26.2; range: 22.7-31.1). Peripheral blood mononuclear cells (PBMCs) were isolated from matched autologous blood samples of the same donors. All primary cells were maintained under standard cell culture conditions (37 °C, 5% CO_2_) and used at low passage numbers to preserve donor-specific phenotypes. No immortalized or established cell lines were used in this study; therefore, formal cell line authentication was not applicable. Cells were routinely tested for mycoplasma contamination using PCR-based assays, which were performed at least monthly and prior to experimental use. All tests were negative.

#### Sex as a biological variable

Sex was recorded for all participants. The clinical cohort was predominantly male, and the *in vitro* cohort consisted exclusively of male donors. As a result, the study was underpowered to assess sex-dependent effects, and potential influences of sex or gender on immune profiles or healing outcomes could not be determined. This limitation is explicitly acknowledged in the limitations of the study section.

### Method details

#### Surgery and rehabilitation

Patients with acute Achilles tendon rupture underwent minimal-invasive Achilles tendon repair using the Dresden instrument as inaugurated by Amlang et al.^63^ In contrast to the original described technique, tendon ends were secured in an over-tightened manner and AT was shortened in maximum plantarflexion as described previously.^57^

Postoperatively, the foot was placed in a walker with 30° of plantarflexion for six weeks. Afterwards, the heel height was gradually reduced over a period of 2 weeks until neutral position was reached. Early functional rehabilitation was initiated with partial weight bearing and physiotherapy. Physiotherapy program was supervised for 12 weeks and consisted of functional training of the muscles and proprioceptive as well as coordinative training. Plantarflexion exercises started after the third week and were limited to neutral position. Each patient received a detailed handout for physiotherapy after surgery.

#### Patient’s outcome analysis

To evaluate the subjective and functional healing outcome of the Achilles tendons, the patients were examined at 6 weeks, 6 months as well as 12 months after surgery in the outpatient clinic. According to a standardized protocol, the following parameters were evaluated: subjective satisfaction score (1-6 points; 6=worst), visual analog scale (VAS) for pain (1-10 points; 10=worst), VAS for function (1-10 points; 10=worst), Achilles tendon Total Rupture Score (ATRS; 1-100; 100=best), and Hannover Score (1-100; 100=best). The ATRS represents a patient-reported outcome measure, capturing subjective symptoms and perceived functional limitations. At the time of recruitment, no validated German version of the ATRS existed; therefore, a translated version was used. The reliability and validity of the German ATRS were later confirmed.^64^ Objective functional parameters included the Matles test for clinical evaluation of Achilles tendon elongation, corresponding to the later described Achilles tendon resting angle (ATRA) measurement.^65^ Matles test was calculated as the plantarflexion angle relative to the contralateral side (higher angle=greater elongation). Additional objective measures included the heel rise test (heights and repetitions relative to contralateral side, high=worst), and the maximum calf circumference (MCC), measured 15 cm below the medial knee joint line and given as relative to pre-operative state in cm, high=worst). Maximum heel-rise height was measured as the vertical distance between the plantar surface of the heel and the floor while the patient stood on one leg in maximal tiptoe position.^66^ To test endurance of the calf muscles, patients performed as many single-leg heel-raises as possible within one minute; if the test was interrupted prematurely, the execution time on the unaffected leg was adjusted accordingly.

#### Magnetic resonance (MRI) imaging and evaluation

MRI was used to assess tendon length, muscle volume (MV) of the musculus soleus and musculus gastrocnemius lateralis/medialis, as well as fatty degeneration (FD) of these muscles. Each patient underwent four MRI examinations of both lower legs (pre-operative, 6 weeks, 6 months and 12 month). The pre-operative MRI served as internal control. All MRI scans were carried out on a 1.5 Tesla system (Magnetom Aera; Siemens Healthcare) using T1-weighted and Dixon sequences. All image analyses were performed by two independent radiological experts blinded to the study results. Tendon length was measured using Osirix Lite (version 7.5.1) from the calcaneal insertion to the musculotendinous junction of the medial gastrocnemius, and expressed as % of the contralateral side. The MV was calculated as % of the pre-operative MV of the affected leg. For FD analysis, a two-point Dixon MRI sequence was applied to separate fat and water signals based on proton resonance frequency shifts. Image post-processing and region-of-interest (ROI) analysis of the m. gastrocnemius medial/lateral and m. soleus were performed using 3D Slicer (version 4.6, Slicer Community, www.slicer.org). FD was calculated as ((*M*_*in*_ − *M*_*op*_) / (2×*M*_*in*_)) × 100, where *M*_*in*_ and *M*_*op*_ denote mean in-phase and opposed-phase signal intensities, respectively. The measured percentage of FD of the muscles is given as fold change to the pre-operative stage of the affected side. MV and FD were calculated both as total (sum of all three muscles) and individually for m. soleus, the muscle most affected in Achilles tendon pathologies. A complete set of MRI follow-up data (pre-operative, 6 weeks, 6 months, and 12 months) were available for 24 of the 26 patients; two patients did not undergo MRI due to scheduling constraints or contraindications.

#### Flow cytometry analysis for correlation with clinical outcome

Human peripheral blood and hematoma aspirate of the Achilles tendon rupture patients were collected in EDTA sampling tubes at the time of surgery and 6 weeks, 6 months and 12 months postoperatively (blood only). White blood cells were isolated from 200 μl of whole blood or all available volume of hematoma aspirate by incubation for 12 minutes with 4 ml of lysis buffer (1x Red Blood Lysis (RBL) Buffer, eBioscience). After centrifugation the resulting cell pellet was washed and resuspended in FACS buffer: phosphate buffered saline w/o Mg/Ca (PBS, Gibco) with 1% fetal calve serum (FCS, Biochrom/Sigma-Aldrich). The cells were stained for the T-cell related surface markers CD3, CD4, CD8, CD11a, CD28, CD57, as well as CD19, CD45 and Live/Dead reagent (Table S3) in FACS-buffer for 25 minutes. After staining, cells were fixed in 1% paraformaldehyde (PFA) solution and measured within one day using the BD Canto II System at the BIH Cytometry Core Facility. Frequency minus one (FMO) controls were performed exemplarily for validation and gating strategy. The gating strategy with FMO controls is depicted in Figure S4. The number of cells is given as percentage of the parent population.

#### Human PBMC and Achilles tendon cell isolation and culture for *in vitro* study

Peripheral blood was taken pre-operatively from the six Achilles tendon rupture patients and collected in Heparin sampling tubes. Peripheral blood mononuclear cells (PBMCs) were directly isolated using SepMate™-50 tubes and Lymphoprep™ medium (both STEMCELL Technologies Inc.) by density gradient centrifugation. Isolated PBMCs were stored in RPMI 1640 (PAN Biotech) supplemented with 60% FCS) and 10% dimethyl sulfoxide (DMSO, Sigma-Aldrich) at -170°C until further use. Tenocytes of the respective donors were isolated by 0.3% collagenase digestion as described previously.^67^ Cells were cultured with tenocyte culture medium (Teno-CM): DMEM/Ham’s F12 (1:1, Sigma-Aldrich) with 10% FCS and 1% penicillin/streptomycin (Sigma-Aldrich) at 37°C, 95% humidity and 5% CO_2_. After reaching 80% confluency, cells were cryopreserved in liquid nitrogen until further use. Mycoplasma testing was routinely performed with cell cultures on a monthly basis to ensure culture integrity and prevent contamination.

#### Polarization and T cell sorting

For polarization, round buttom 96-well plates (Greiner Bio-One) were coated with anti-human CD3 antibody (BD Bioscience) in PBS w/o Mg/Ca and incubated at 4°C overnight. Next day, PBMCs were thawed and seeded into coated 96-well plates with X-Vivo™ 15 medium (Lonza) supplemented with anti-human CD28 antibody (BD Bioscience) and the respective agents to polarize T cells either into IFNG, or IL17 produces, or left them unpolarized for four days. Polarizations were performed according to a modified protocol of Delens et al. for IL17 production using 25 ng/ml recombinant human (rhu) IL6, 6.25 ng/ml rhuTGFB1, 12.5 ng/ml rhuIL1B (all Peprotech), 25 ng/ml rhuIL23 (R&D), and 500 ng/ml anti-human IFNG antibody (BD Bioscience).^68^ IFNG polarization was induced with 20 ng/ml rhuIL12p70 (Peprotech), 5 ng/ml rhuIL2 (Novartis), and 500 ng/ml anti-human IL4 antibody (BioLegend). After T cell polarization, the polarization medium (Pol-M) of the respective groups were collected and unpolarized CD4^+^ and CD8^+^ T cells, as well as IFN-polarized CD8^+^ and IL17-polarized CD4^+^ T cells prepared for sorting. Therefore, 1x10^6^ cells were incubated with Live/Dead Fixable (L/D, Invitrogen) in 100 μl PBS w/o Ca/Mg in the dark at 4°C for 30 minutes. Afterwards, cells were stained with antibodies against CD45, CD3, CD4, CD8 and TCRγδ (Table S3) diluted in PBS w/o Ca/Mg with 2% FCS and incubated at 4°C for 15 minutes. After staining, cells were sorted into CD4^+^ or CD8^+^ T cells using the cell sorter BD FACS Aria II by the BIH Cytometry Core Facility (Berlin, Germany) and subsequently used for coculture with autologous tenocytes.

#### Wound healing assay

Tenocytes were seeded with 2.5x10^4^ cells/ml Teno-CM into 24-well plates and cultured at 37°C until reached 100% confluence. A wound (scratch) was induced using a 100 μl pipette tip and wells were scanned with the Tecan SPARK® multimode microplate reader (Tecan). Afterwards, 2.5x10^4^ of the sorted CD4^+^ or CD8^+^ T cells of the respective polarization groups in 1 ml of the experimental medium (Exp-M: ½ Teno-CM, ¼ X-Vivo™, ¼ Pol-M IFNG, IL17 or Unpol) were added to the tenocytes. Tenocytes without T cells with Teno-CM served as untreated control. All conditions were prepared in triplicates. Cultivation was done at 37°C for 45 hours. At time points 0, 15, 25, and 45 hours the wound healing was documented with the Tecan SPARK® scanner and individual wells with the microscope (10x, Leica). At earlier time points (15 and 25 hours), no washing was performed, and T cells remained present during imaging. At the final 45-hour time point, a five-time washing step with PBS was performed to remove non-adherent T cells before imaging, allowing accurate area quantification. The wound closure was measured using ImageJ (version 1.53o). At 45h, supernatants were also collected and stored at –20 °C for further analysis, and cells were lysed immediately for RNA isolation.

#### Cell contraction assay

A cell suspension with 7x10^4^ tenocytes and 3.5x10^4^ sorted CD4^+^ or CD8^+^ T cells of the respective polarization groups per gel was prepared in Exp-M IFNG, IL17 or Unpol. Tenocytes without T cells in Teno-CM served as untreated control. Gel suspension was prepared on ice with 1.5x PBS (10x Biochrom), 6.4 mM sodium hydroxide (Sigma-Aldrich), 1.54 mg/ml Collagen Typ I (rat tail tendon, Corning) ad 250 μl ddH_2_O per gel. A total of 250 μl gel suspension was added to 100 μl cell suspension and transferred into self-manufactured silicone dishes rings (1 cm inner diameter) placed in a 12-well plate (BD Falcon). The coculture gels were prepared in duplicates. After 30 minutes incubation at 37°C, 1 ml of the respective Exp-M or Teno-CM was added to each well and silicone rings removed carefully to release the polymerized gels containing the tenocyte/T-cell coculture. The gels were incubated for 3 days and gel contraction documented at time points 0, 15, 25, 45 and 65 hours. Reduction of gel area was analyzed using ImageJ (Version 1.53o, public domain).

#### Gene expression analysis

RNA was isolated from the triplicates of the cell migration experiment using the NucleoSpin® RNA isolation mini kit (Macherey Nagel) according to the manufacturer instructions. The RNA was quantified with the NanoDrop™ 1000 spectrophotometer (PeqLab Biotechnologie) and afterwards stored at -80°C. A total of 100 ng RNA was transcribed to cDNA with the qScript™ cDNA synthesis SuperMix (Quanta Biosciences). Quantitative Real-Time PCR (qRT-PCR) was performed with the PerfeCTa® SYBR® Green SuperMix (Quanta Biosciences) according to the manufacturer and using the Light Cycler 480 System (Roche). Primer sequences listed in Table S4 were designed using Primer 3 software (Freeware; available online: http://frodo.wi.mit.edu/primer3) and produced by Tib Molbiol. All primers were tested for amplification efficiency and an efficiency correct equation was used to calculate the normalized gene expression to the reference gene hypoxanthine phosphoribosyl transferase (HPRT), which was tested to be the most constant housekeeping gene.

#### Protein analysis

Protein concentrations of IFNG and IL17 was measured for supernatants of the PBMC polarization to verify that IFNG and IL17 polarization has worked. MMP1, MMP2, MMP3, and TIMP1 concentrations were quantified in supernatants from the migration assay (45 hours time point). All proteins were quantified by DuoSet® ELISA Kits (R&D Systems) for human IFNG, human IL17, human MMP1, 2, 3 and human TIMP1. To achieve sample concentrations, which fit in the range of the standard curve, samples were diluted accordingly to the used ELISA kit with 1x Reagent Diluent (DuoSet® Kit). The assays were performed in accordance to the instructions of the manufacturer and absorbance measured with the Tecan Infinite Pro® multiplate reader (Tecan).

### Quantification and statistical analysis

Statistical analysis was performed using GraphPad Prism (version 7.0, GraphPad Software). To evaluate a relationship between the biology at the tendon rupture side at the time of surgery or the peripheral blood status and the patient outcome after 6 and 12 months, Spearman correlation analysis was performed. Patients were grouped according to the ATRS in “successful healer” (ATRS≥90 points) and “poor healer” (ATRS<70). Patients with and without tendon elongation were identified by Matles test (elongation: >7 degree (n=15); non-elongation: <4 degree (n=7)) and evaluated regarding the impact of CD8^+^ T cell subpopulations. Mann-Whitney-U Test was performed to analyze significant differences between the groups. ROC analysis with 95% confidence interval was performed to evaluate the prognostic value of T cells with a cut-off for healers and non-healers at ATRS of 70 points and tendon elongation versus non-elongation at Matles test of 7 degree. For the *in vitro* proof of mechanism, statistical differences were analyzed using Friedman tests and Wilcoxon matched-pairs test for paired samples. P < 0.05 was considered as statistically significant.
