# Supplementary material for: CD4+ to CD8+ T cell imbalance drives poor Achilles tendon repair in patients
Source: iScience. 2026 Jan 2;29(2):114612. doi: 10.1016/j.isci.2025.114612 (PMC12857407; doi:10.1016/j.isci.2025.114612)
Supplement: Document S1. Figures S1–S4 and Tables S1–S4 [file mmc1.pdf]

iScience, Volume *29*

Supplemental information

CD4^{+} to CD8^{+} T cell imbalance drives poor Achilles tendon repair in patients

Franka Klatte-Schulz, Sven Geißler, Nicole Bormann, Susann Minkwitz, Serafim Tsitsilonis, Sebastian Manegold, Tobias Gehlen, Josephine A. Melzer, Alper Kurtoglu, Aysha Bonell, Katharina Schmidt-Bleek, Georg N. Duda, Birgit Sawitzki, and Britt Wildemann

1. Supplementary Figures

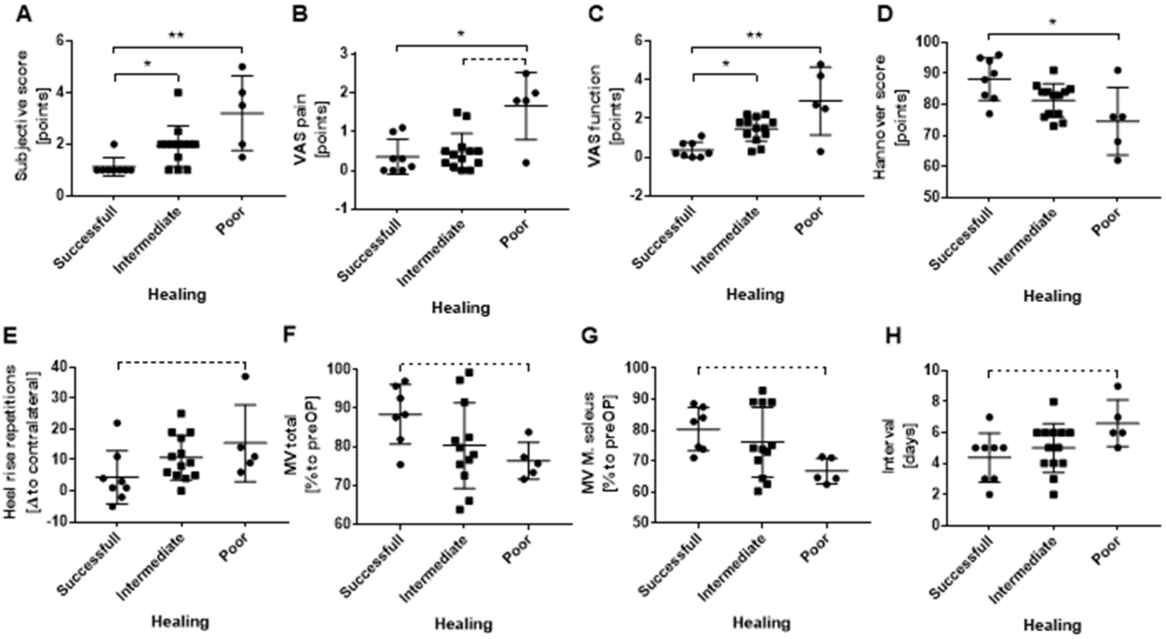

Fig. S1: Grouping of successful, intermediate, and poor healing patients according to the ATRS (>90, <90>70, <70). (A) Subjective score: 0-6, with 6 being worst; (B/C) VAS pain/function: 0-10, with 10 being worst; (D) Hannover score: 0-100, with 100 being best; (E) Δ heel rise repetitions: measured as difference to the contralateral side; (F) total MV: calculated as sum of the three muscles m. soleus, m. gastrocnemius lateralis/medialis and given in % to the pre-operative state; (G) MV of solely m. soleus given in % to pre-operative state; (H) Interval: time from rupture to surgery. n=8 successful healer, n=13 intermediate healer, n=5 poor healer. Statistics: Kruskal Wallis Test and Dunn's multiple comparisons test, p≤0.05 (*), p≤0.01 (**). A dashed line indicates a trend (≤0.1).

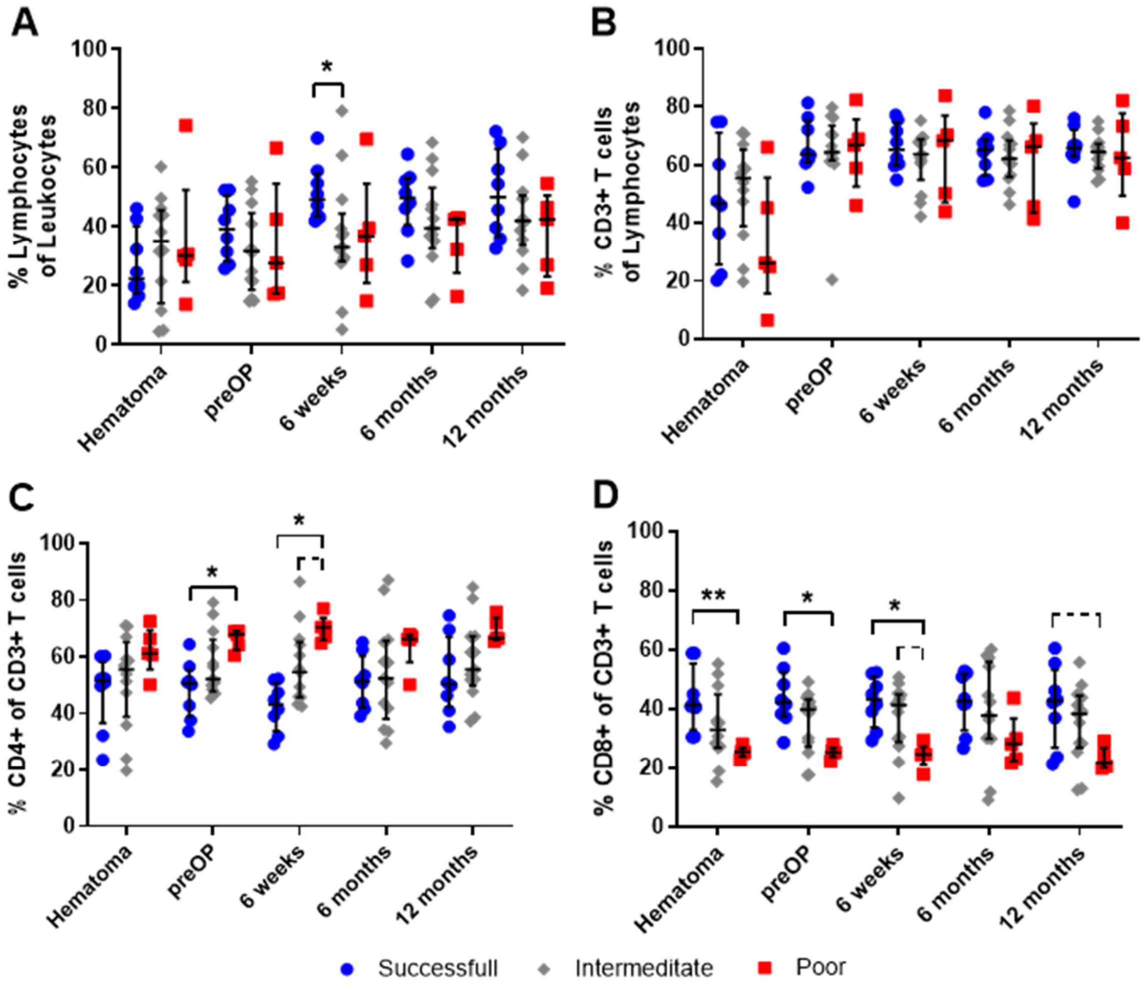

Fig. S2: T cells in Achilles tendon healing outcome: (A) Lymphocytes, (B) CD3+, (C) CD4+, and (D) CD8+ T cells in successful, intermediate and poor healing group according to the ATRS (>90, <90>70, <70) after 12 months. Cell populations were measured by flow cytometry and given in % to the respective parent population. n=8 successful healer, n=13 intermediate healer, and n=5 poor healer, Statistics: Kruskal Wallis Test and Dunn's multiple comparisons test, p≤0.05 (*), p≤0.01 (**). A dashed line indicates a trend (≤0.1).

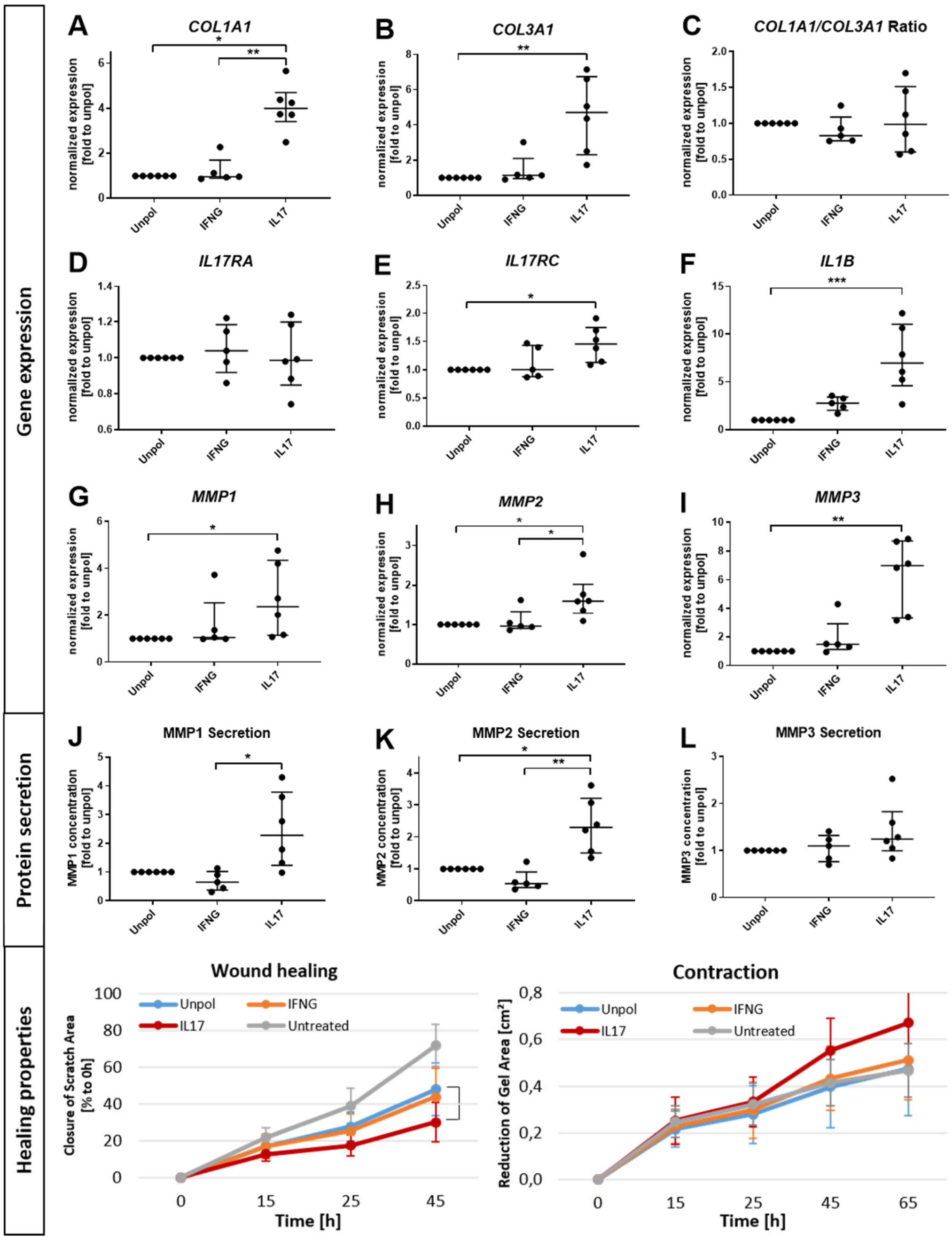

Fig. S3: Gene expression and matrix remodeling in tenocytes stimulated with conditioned medium (CM) from polarized and unpolarized T cells. Tenocytes were cultured in medium conditioned from unpolarized (Unpol), IL17–polarized, or IFN-polarized cells for 45 h. (A–I) Gene expression levels were normalized to the housekeeping gene HPRT and expressed as fold change relative to unpolarized CM (n = 5–6). (J–L) Protein secretion is shown as fold change to unpolarized CM (n = 5–6). IL17–CM increased *COL1A1*, *COL3A1*, *IL17RC*, *IL1B*, *MMP1*, *MMP2*, and *MMP3* expression, while IFN-CM had neutral effects. Statistics: Kruskal–Wallis test with Dunn's multiple comparisons; $p \leq 0.05$ (*), $p \leq 0.01$ (**), $p \leq 0.001$ (***).

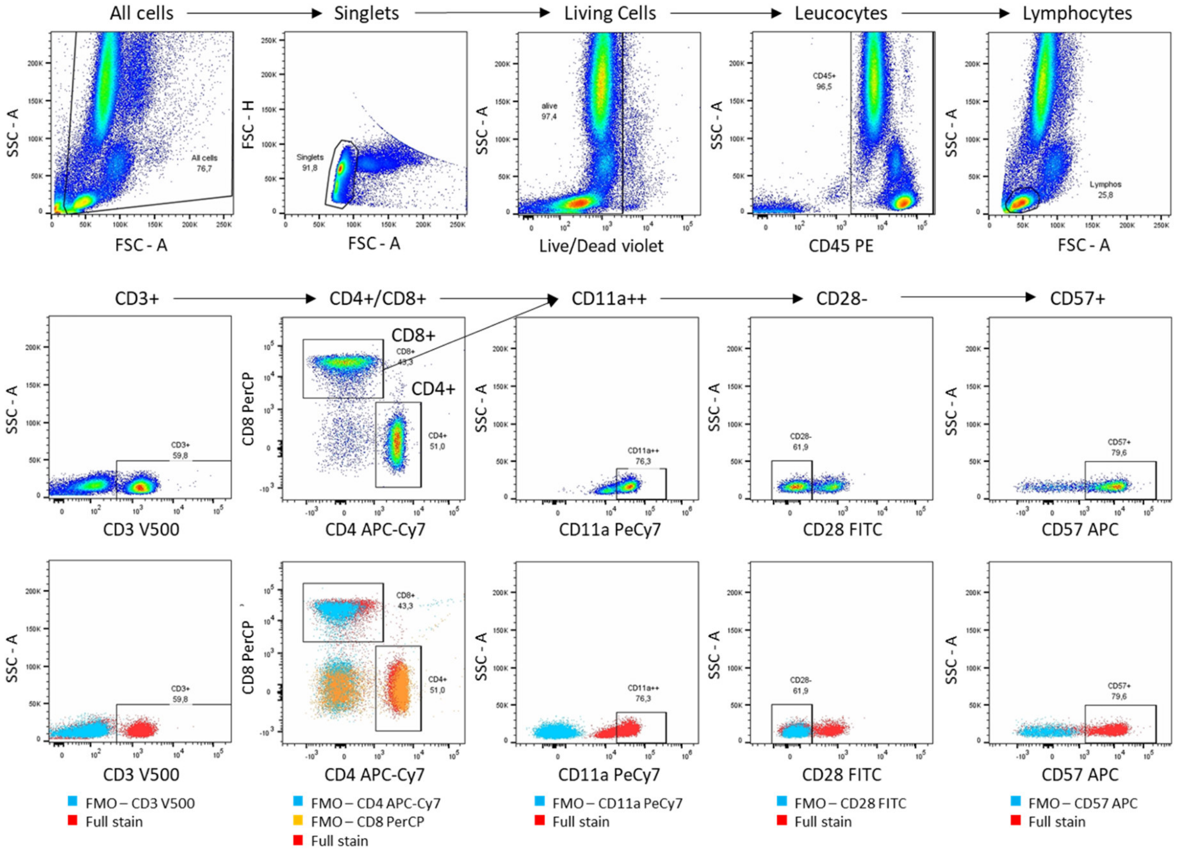

Fig. S4: Flow cytometry gating strategy and FMO controls. CD4+ and CD8+ T cells were assessed as percentage of CD3+ lymphocytes. CD11a++/CD28-/CD57+ memory T cells were evaluated as percentage of CD8+ T cells. Frequency minus one (FMO) controls (last row) verify gate settings.

2. Supplementary Tables

Table S1: Distribution of cell populations in hematoma aspirate and peripheral blood

Mean percentage ± StDev	Hematoma aspirate	Peripheral blood			
		preOP	6 weeks	6 months	12 months
**n-value**	28	31	27	27	26
**Interval [days] (Rupture – Surgery)**	4.8 ± 1.7	4.9 ± 1.7	5.1 ± 1.7	5.1 ± 1.7	5.1 ± 1.7
**Age [years]**	39.5 ± 10.3	39.4 ± 10.0	39.5 ± 10.1	39.5 ± 10.1	39.1 ± 10.0
**BMI**	24.8 ± 2.4	24.8 ± 2.3	24.9 ± 2.1	24.9 ± 2.1	24.8 ± 2.0
**Cell populations [%]**					
**Leukocytes (CD45+)**	96.5 ± 3.5	96.9 ± 3.2	97.2 ± 4.1	94.5 ± 8.5	95.7 ± 4.3
**Lymphocytes**	32.1 ± 16.4	33.9 ± 14.1 *12 mon	40.8 ± 17.9	41.8 ± 14.5	44.4 ± 14.8
**T cells (CD3+)**	45.9 ± 20.3	66.4 ± 12.0 *hema	62.8 ± 11.0	62.6 ± 10.0	64.3 ± 9.0
**T helper cells (CD4+)**	54.5 ± 12.9	57.0 ± 11.3	57.7 ± 12.0	55.6 ± 14.5	59.1 ± 13.6
**Cytotoxic T cells (CD8+)**	35.8 ± 12.0	35.8 ± 10.8	36.0 ± 11.2	37.7 ± 13.7	34.6 ± 13.3
**Memory T cells (CD8+CD11a++)**	57.8 ± 15.0	61.7 ± 15.0	58.1 ± 15.5	64.4 ± 15.0	62.8 ± 14.1
**CD28- memory T cells (CD8+CD11a++CD28-)**	33.0 ± 15.7	25.3 ± 14.7 *hema, 6, 12 mon	27.6 ± 14.6	32.3 ± 16.4	31.6 ± 14.9
**CD57+ memory T cells (CD8+CD11++CD57+)**	32.5 ± 14.5	26.2 ± 13.7 *hema, 6, 12 mon	27.9 ± 12.9	30.7 ± 13.3	31.1 ± 14.1
**CD28-CD57+ memory T cells (CD8+CD11a++CD28-CD57+)**	26.8 ± 13.6	20.0 ± 13.2 *hema, 6, 12 mon	22.4 ± 12.5	25.5 ± 13.3	25.2 ± 13.4

*: significant differences between cell populations in pre-operative peripheral blood versus hematoma aspirate as well as peripheral blood over time. Friedman test for paired samples, $p \leq 0.05$.

Table S2: Correlations between T cells and clinical outcome scores after 12 months

	r_s / p	Hematoma aspirate	Peripheral blood			
			preOP	6 weeks	6 months	12 months
T cell populations	**CD3+**	Matles: - 0.398/0.049				
	**CD4+**	Subj. score: +0.500/0.015<br>VAS fct: +0.419/0.046<br>Total MV: -0.483/0.023<br>MCC: -0.432/0.040	Subj. score: +0.433/0.027<br>VAS fct: +0.453/0.017<br>ATRS: -0.488/0.012<br>Hannover: -0.394/0.046<br>Heel Rise Reps: +0.435/0.026<br>Total MV: -0.586/0.003<br>MV m.sol: -0.425/0.039<br>MCC: -0.507/0.008	VAS pain: +0.405/0.040<br>VAS fct: +0.514/0.007<br>Matles: +0.526/0.006<br>ATRS: -0.443/0.023	Matles: +0.451/0.021	VAS pain: +0.471/0.015<br>Matles: +0.535/0.005
	**CD8+**	Subj. score: -0.608/0.002<br>VAS pain: -0.432/0.040<br>VAS fct: -0.577/0.004<br>ATRS: +0.545/0.007<br>Total MV: +0.499/0.018<br>MV m.sol: +0.428/0.047<br>FD m.sol: -0.428/0.047<br>MCC: +0.439/0.036	Subj. score: -0.461/0.018<br>VAS fct: -0.527/0.006<br>Matles: -0.428/0.029<br>ATRS: +0.534/0.005<br>Total MV: +0.534/0.007	Subj. score: 0.407/0.039<br>VAS pain: -0.413/0.036<br>VAS fct: -0.498/0.010<br>Matles: -0.545/0.004<br>ATRS: +0.438/0.012	Matles: -0.480/0.013	VAS pain: -0.428/0.029<br>Matles: -0.590/0.002
	**CD8+CD11a++**			Matles: -0.425/0.030		
	**CD8+CD11a++ CD28-**			Matles: -0.598/0.001	Matles: -0.488/0.011	Matles: -0.481/0.013
	**CD8+CD11a++ CD57+**			VAS pain: -0.404/0.041<br>Matles: -0.510/0.008	Matles: -0.471/0.016	Matles: -0.399/0.044
	**CD8+CD11a++ CD28-CD57+**			Matles: -0.573/0.002	Matles: -0.469/0.016	VAS fct: -0.435/0.026<br>Matles: -0.420/0.032

Subj. score (subjective score): 1-6, 6=bad; VAS pain/fct (visual analog scale pain/function): 1-10, 10=bad; Matles: Achilles tendon resting angle in plantarflexion relative to contralateral side, high=bad; ATRS (Achilles tendon total rupture score): 1-100, 100=best; Hannover: 1-100, 100=best; heel rise reps (heel rise repetitions): Δ to contralateral side, high=bad; MCC (maximum calf circumference): relative to pre-operative state in cm, high=bad; MV total/m.sol (muscle volume total/m.soleus): % to pre-operative muscle volume of affected leg, high=good; FD m.sol (fatty degeneration m.soleus): fold to pre-operative stage of the affected side: high=bad

Table S3: Antibodies for flow cytometry and cell sorting

AK	Fluorochrome	Clone	Company	Cat #	Dilution
	**Flow Cytometry**				
**CD3**	**V500**	SP34-2	BD Horizon	560772	50
**CD4**	**APC Cy7**	RPA-T4	BD Bioscience	561839	50
**CD8**	**PerCP**	SK1	Biolegend	344708	50
**CD11a**	**PECy7**	HI111	BD Bioscience	561387	50
**CD19**	**PE**	HIB19	BD	555413	10
**CD28**	**FITC**	CD28.2	BD	555728	5
**CD45**	**PE**	HI30	eBioscience	12-0459-41	100
**CD57**	**APC**	NK-1	BD Bioscience	560845	1000
**L/D**	**violet**	-	Invitrogen	L34955	1000
**Fluorescence Activated Cell Sorting**					
**CD3**	**BV711**	OKT3	BioLegend	317328	40
**CD4**	**PE**	OKT4	BioLegend	317410	100
**CD8**	**BV605**	SK1	BioLegend	344742	50
**CD45**	**PB**	J33	Beckman	A74763	100
**TCRγδ**	**FITC**	B1	BioLegend	331208	600
**L/D**	**Far Red**	-	Invitrogen	L34973	5000

PE: Phycoerythrin, APC: Allophycocyanin, PerCP: Peridinin Chlorophyll Protein, FITC: Fluorescein Isothiocyanat; BV: Brilliant violet, PB: Pacific Blue; L/D: Live Dead reagent

Table S4: Primer sequences from determined genes (F: forward, R: reverse)

Gene	Accession No.	Primer sequence
HPRT	NM_000194	F: 5’ GAA GGT GAA GGT CGG AGT C 3’<br>R: 5’ GAA GAT GGT GAT GGG ATT TC 3’
COL1A1	NM_000088.3	F: 5’ TGA CCT CAA GAT GTG CCA CT 3’<br>R: 5’ ACC AGA CAT GCC TCT TGT CC 3’
COL3A1	NM_000090.3	F: 5’ AGC CTG GTA AGA ATG GTG CC 3’<br>R: 5’ TCC TTG CCA TCT TCG CCT TT 3’
MMP1	NM_002421.3	F: 5’ CAC GCC AGA TTT GCC AAG AG 3’<br>R: 5’ GTC CCG ATG ATC TCC CCT GA 3’
MMP2	NM_004530	F: 5’ TGG ATG ATG CCT TTG CTC GT 3’<br>R: 5’ CCA GGA GTC CGT CCT TAC CG 3’
MMP3	NM_002422.3	F: 5’ TGG GCC AGG GAT TAA TGG AG 3’<br>R: 5’ GGC CAA TTT CAT GAG CAG CA 3’
TIMP1	NM_003254.2	F: 5’ TTG GCT GTG AGG AAT GCA CA 3’<br>R: 5’ AAG GTG ACG GGA CTG GAA GC 3’
IL6	NM_000600	F: 5’ TGA GGA GAC TTG CCT GGT GA 3’<br>R: 5’ TTG GGT CAG GGG TGG TTA TT 3’
IL1B	NM_000576	F: 5’ TCC AGG AGA ATG ACC TGA GC 3’<br>R: 5’ GTG ATC GTA CAG GTG CAT CG 3’
IL17RA	NM_001289905.1	F: 5’ GCC CAG ACC AGA AGA GTT CC 3’<br>R: 5’ TGA GGC AGT CAT TGA GGC AG 3’
IL17RC	NM_001203265.1	F: 5’ TTT GGT CAG TCT GTG GGC TC 3’<br>R: 5’ GTT CCT TCT CGT ACC TGG GC 3’

HPRT = hypoxanthine phosphoribosyl transferase, Col = collagen, MMP = matrix metalloproteinase, TIMP = tissue inhibitor of metalloproteinase, IL = interleukin.
